# Characterization of fatty acid desaturases reveals stress-induced synthesis of C18 unsaturated fatty acids enriched in triacylglycerol in the oleaginous alga *Chromochloris zofingiensis*

**DOI:** 10.1186/s13068-021-02037-2

**Published:** 2021-09-17

**Authors:** Tao Wu, Lihua Yu, Yu Zhang, Jin Liu

**Affiliations:** Laboratory for Algae Biotechnology & Innovation, College of Engineering, Peking University, Beijing, 100871 China

**Keywords:** Biofuels, Fatty acid desaturation, Green algae, Lipid metabolism, Neutral lipids, Stress induction

## Abstract

**Background:**

The green microalga *Chromochloris zofingiensis* is capable of producing high levels of triacylglycerol rich in C18 unsaturated fatty acids (UFAs). FA desaturation degree is regulated by FA desaturases (FADs). Nevertheless, it remains largely unknown regarding what FADs are involved in FA desaturations and how these FADs collaborate to contribute to the high abundance of C18 UFAs in triacylglycerol in *C. zofingiensis*.

**Results:**

To address these issues, we firstly determined the transcription start sites of 11 putative membrane-bound FAD-coding genes (*CzFAD*s) and updated their gene models. Functional validation of these CzFADs in yeast and cyanobacterial cells revealed that seven are bona fide FAD enzymes with distinct substrates. Combining the validated functions and predicted subcellular compartments of CzFADs and the FA profiles of *C. zofingiensis*, the FA desaturation pathways in this alga were reconstructed. Furthermore, a multifaceted lipidomic analysis by systematically integrating thin-layer chromatography, gas chromatography–mass spectrometry and liquid chromatography–mass spectrometry techniques was conducted, unraveling profiles of polar membrane lipids in *C. zofingiensis* and major desaturation steps occurring in these lipids. By correlating transcriptional patterns of *CzFAD* genes and changes of lipids upon abiotic stress conditions, our results highlighted collaboration of CzFADs for C18 UFA synthesis and supported that both de novo FA synthesis and membrane lipid remodeling contributed C18 UFAs to triacylglycerol for storage.

**Conclusions:**

Taken together, our study for the first time elucidated the pathways of C18 FA desaturations and comprehensive profiles of polar membrane lipids in *C. zofingiensis* and shed light on collaboration of CzFADs for the synthesis and enrichment of C18 UFAs in triacylglycerol.

**Supplementary Information:**

The online version contains supplementary material available at 10.1186/s13068-021-02037-2.

## Introduction

Fatty acids (FAs) are building blocks of acyl-lipids that generally comprise polar membrane lipids (PMLs) and neutral storage lipids. PMLs, serving as cell boundaries, not only maintain the integrity and identity of subcellular compartments, but also play roles in the initiation of intracellular signals [1–3]. On the other hand, neutral storage lipids particularly triacylglycerol (TAG), the most energy-dense lipid class, are important reservoirs for carbon and energy storage [4]. FAs vary in the carbon-chain length and number of double bonds depending greatly on organisms. FAs without double bond are referred to as saturated FAs (SFAs) while those with double bond(s) are designated as unsaturated FAs (UFAs). UFAs can be further classified as monounsaturated FAs (MUFAs; with one double bond) and polyunsaturated FAs (PUFAs; with more than one double bond). MUFAs particularly C18:1^∆9^ are considered as preferred components for making biodiesel with balanced low-temperature and oxidative stability properties [5]. PUFAs especially the ω3 very long-chain ones such as eicosapentaenoic acid (EPA, 20:5^Δ5,8,11,14,17^) and docosahexaenoic acid (DHA, 22:6Δ^4,7,10,13,16,19^), on the other hand, have long been used as value-added nutraceuticals with potent beneficial effects on human health [6]. The desaturation degree of FAs, determined by the action of FA desaturases (FADs), varies across organisms and responds to both abiotic and biotic stresses [7–10]. Understanding function and physiological roles of FADs helps to facilitate genetic engineering of FA composition for intended purposes.

The functional roles of FADs have been well studied in land plants particularly the model plant *Arabidopsis thaliana*, which harbors over ten FAD-coding genes [9, 10]. In Arabidopsis, de novo FA synthesis occurs in the chloroplast leading to formation of C16:0 and C18:0 thioesterified with the acyl carrier protein (ACP). AtFAB2, a stearoyl-ACP desaturase (SAD) localized in the chloroplast stroma, is soluble and catalyzes the formation of C18:1^∆9^ from C18:0 [11, 12]. C16:0 and C18:1^∆9^ can be either incorporated into the chloroplast membrane lipids or exported out of the chloroplast and then incorporated into the endoplasmic reticulum (ER) membrane lipids for further desaturation. AtFAD4 and AtFAD5, both residing in the chloroplast, act on C16:0 in phosphatidylglycerol (PG) to form C16:1^∆3t^ (a *trans* double bond on the ∆3 position) [13] and on C16:0 in monogalactosyldiacylglycerol (MGDG) to form C16:1^∆7^ [14, 15], respectively. AtFAD2 is an ER-located ∆12 desaturase and functions in converting C18:1^∆9^ to C18:2^∆9,12^ [16, 17]. AtFAD6, on the other hand, is a chloroplast-targeted ω6 desaturase structurally unrelated to AtFAD2 and can desaturate C18:1^∆9^ to C18:2^∆9,12^ and C16:1^∆7^ to C16:2^∆7,10^ in the chloroplast membrane lipids [18, 19]. To produce the ω3 FAs C16:3^∆7,10,13^ and C18:3^∆9,12,15^, ω3 desaturases are needed. Arabidopsis has three isozymes of ω3 desaturase, AtFAD3, AtFAD7 and AtFAD8. AtFAD3 is localized in the ER and desaturates C18:2^∆9,12^ in ER membrane lipids to C18:3^∆9,12,15^ [20]. By contrast, AtFAD7 and AtFAD8 reside in the chloroplast and act on both C18:2^∆9,12^ and C16:2^∆7,10^ in the chloroplast membrane lipids to form C18:3^∆9,12,15^ and C16:3^∆7,10,13^, respectively [21, 22].

Similar to Arabidopsis, algae particularly green microalgae perform the de novo FA synthesis in the chloroplast and carries out FA desaturations in both compartments of the chloroplast and ER [6, 23]. The model green alga *Chlamydomonas reinhardtii*, with annotated chromosome-level genome sequence and well-developed genetic tools, has long been used to study lipid metabolism including FA desaturation [23]. Similarly, CrSAD is believed to catalyze the desaturation of C18:0-ACP in the chloroplast of *C. reinhardtii* to form C18:1^∆9^-ACP, which is either incorporated into chloroplast membrane lipids or released as free FA and then exported outside the chloroplast for ER lipids. CrFAD2 is responsible for the desaturation of C18:1^∆9^ in ER membrane lipids while CrFAD6 likely functions as a ω6 desaturase in the chloroplast. Nevertheless, *C. reinhardtii*, unlike Arabidopsis, contains only one ω3 desaturase (i.e., CrFAD7), which is located in the chloroplast; and upon *CrFAD7* suppression, more ω6 FAs (both C16 and C18) accumulate at the expense of ω3 FAs [24]. Probably, the envelop localization characteristic allows CrFAD7 to access both chloroplast lipids and ER lipids through the chloroplast–ER contact site, for the formation of ω3 FAs. It is also possible that the ω3 FAs are produced entirely in the chloroplast and then exported out of the chloroplast for ER membrane lipids. As *C. reinhardtii* synthesizes FAs (e.g., C16:4^∆4,7,10,13^ and C18:3^∆5,9,12^) that are not present in Arabidopsis, additional FADs are involved, including Cr∆4FAD that is chloroplast-located and introduces a double bond on the ∆4 position of C16:3^∆7,10,13^ in MGDG [25], and CrDES that is a front-end ω13 desaturase and introduces a double bond on the ∆5 positions of C18 UFAs in the ER membrane lipids [26].

Nevertheless, *C. reinhardtii* is generally not considered as a good production strain as the biomass density that can be achieved for this alga is unsatisfactory. By contrast, *Chromochloris zofingiensis*, a unicellular green alga closely related to *C. reinhardtii*, is able to grow robustly under multiple trophic conditions and achieve ultrahigh biomass densities under the heterotrophic fed-batch mode using glucose as the carbon source [27–30]. Moreover, *C. zofingiensis* has the ability to synthesize high levels of triacylglycerol (TAG) rich in C18 UFAs [28, 31, 32], which is ideal for making biodiesel of high quality. In addition to TAG, the alga is capable of producing astaxanthin [33], a value-added keto-carotenoid with a broad range of applications in food, feed, nutraceutical and pharmaceutical industries [34–36]. The concurrent synthesis of TAG and astaxanthin in *C. zofingiensis* [28, 30, 37–39] allows integrated production of these two compounds, which has the potential to offset the algae-based biodiesel production cost. Furthermore, thanks to the availability of chromosome-level genome sequence [40], workable random mutagenesis [40–43], and increasing transcriptomics data [44–47], *C. zofingiensis* has been cited as an emerging model alga for studying lipid metabolism [48].

Although the FA composition of *C. zofingiensis* has long been profiled, what FADs are involved in FA desaturations, what substrates these FADs use, and how they respond to environmental fluctuations and contribute to high-abundance C18 UFAs in TAG remain largely unknown. *C. zofingiensis* genome is predicted to encode 13 putative membrane-bound FADs [40]. Here, we firstly determined the full-length coding sequences of these putative *FAD* genes and confirmed that the 13 genes in fact belong to 11 gene loci that encode membrane-bound FADs of six subgroups. To better understand their functional roles, we then conducted a multifaceted study by integrating the functional validation in yeast and cyanobacterial cells, transcriptional profiling of *FAD* genes and lipidomics of *C. zofingiensis* responding to various abiotic stress conditions. Our study reconstructed the pathways of C18 FA desaturations, profiled the polar membrane lipids and provided implications into the collaboration of FADs for C18 FA synthesis and enrichment in TAG in *C. zofingiensis*.

## Results

### Identification of *CzFAD* genes and bioinformatics analysis

Using the FADs from *C. reinhardtii* and Arabidopsis as the query sequences, Blast against the *C. zofingiensis* non-redundant protein sequences database revealed the presence of 14 putative FAD-coding genes (Additional file 2: Table S1). All except *CzSAD* that encodes a soluble desaturase [49] remain to be characterized. As Cz11g21120 and Cz11g21110 have much shorter coding sequences than the regular *FAD*s and are adjacently located in the same chromosome, they may belong to the same gene locus, but were annotated as two independent ones. In support of this, while producing no fragment using the primers CzFAD6B-pYES2/CT-F1 and CzFAD6B-pYES2/CT-R1 (Additional file 2: Table S2) located at the start and stop codons of Cz11g21120 (Additional file 1: Figure S1), reverse-transcription PCR amplification using CzFAD6B-pYES2/CT-F and CzFAD6B-pYES2/CT-R2 (Additional file 2: Table S2) located at the start codon of Cz11g21120 and the stop codon of Cz11g21110, respectively (Additional file 1: Figure S1), gave rise to a single product that was revealed by Sanger sequencing to contain both coding sequences of Cz11g21120 and Cz11g21110. Similarly, Cz12g10220 and Cz12g10230 belong to the same gene locus, but were annotated as two adjacent ones (Additional file 1: Figure S1). In this context, *C. zofingiensis* harbors 11 rather than 13 putative membrane-bound FAD-coding genes, which are named as *CzFAD2*, *CzFAD6A*, *CzFAD6B*, *CzFAD7A*, *CzFAD7B*, *CzFAD5A*, *CzFAD5B*, *CzFAD5C*, *CzFAD3A*, *CzFAD3B* and *CzFAD4* (Additional file 2: Table S1).

The 5′ RACE experiment was performed to determine the transcriptional start sites of the 11 *CzFAD* genes (Additional file 1: Figure S2a), using the primers designed from their known coding sequences (Additional file 2: Table S3). The 5′ untranslated region (5′ UTR) of these genes ranged from 41 (*CzFAD7A*) to 442 bp (*CzFAD6B*) (Additional file 2: Table S1). Based on the 5′ UTR and stop codon sequences, primers (Additional file 2: Table S2) were designed to amplify the full-length coding sequences (Additional file 1: Figure S2b), which, ranging from 1,092 to 1,821 bp, were validated by Sanger sequencing and deposited in NCBI Genbank with accession numbers shown in Additional file 2: Table S1. Comparison between gene models of *CzFADs* annotated by Roth et al. [40] and our confirmed ones revealed that the previous gene models for *CzFAD2*, *CzFAD6B*, *CzFAD5A* and *CzFAD4* were incomplete(Additional file 1: Figure S1).

Based on the protein sequences deduced from the full-length coding sequences, conserved domain prediction using the software CDD of the NCBI website showed that all the 11 CzFADs except CzFAD4 contain the membrane FA desaturase-like domain (Additional file 1: Figure S3). Besides, CzFAD3A and CzFAD3B harbor an N-terminal cytochrome b5-like heme/steroid binding domain, which is widely present in the front-end desaturases of eukaryotic species including algae, protozoa, fungi, plants and animals [50]. CzFAD4, on the other hand, contains a THEM189_B domain (Additional file 1: Figure S3), which is also found on the desaturase FAD4 (AtFAD4) from Arabidopsis [13]. Protein sequence alignment showed that all CzFADs except CzFAD6B possess three conserved histidine boxes, H(X)_3-4_H, H(X)_2-3_HH and H/Q(X)_1-2_HH (Additional file 1: Figure S4), which bind the two iron ions and are critical for the desaturation activity of FADs [8]. Transmembrane domain prediction suggested that all the 11 CzFADs contain transmembrane domains (Additional file 1: Figure S5). Subcellular localization prediction by Predalgo, a specific program trained on *C. reinhardtii* and suitable for green algae [51], indicated that CzFAD6A, CzFAD7, CzFAD5A, CzFAD5C, CzFAD3A and CzFAD4 are targeted to the chloroplast, while CzFAD2, CzFAD6B, CzFAD5B and CzFAD3B are localized outside the chloroplast likely in the endoplasmic reticulum (ER) (Additional file 2: Table S1). Nevertheless, to validate these subcellular predictions, future experiments are needed.

To understand the evolutionary position of CzFADs, a phylogenetic analysis was conducted using FADs of different functions from various organisms (Additional file 1: Figure S6). These FADs are roughly clustered into nine groups: CzFAD2 falls in the group I of ER ∆12 FADs containing *C. reinhardtii* FAD2 (CrFAD2) that functions in converting C18:1^∆9^ to C18:2^∆9,12^ [52] and Arabidopsis FAD2 (AtFAD2) that acts on extraplastidial C18:1^∆9^ and introduces a double bond on the ∆12 position [53, 54]; CzFAD6A and CzFAD6B are in the group IV of plastidial ω6 FADs including CrFAD6 and AtFAD6 that desaturates not only C18:1^∆9^ to C18:2^∆9,12^ but also C16:1^∆7^ to C16:2^∆7,10^ [18, 19, 55, 56]; CzFAD7A and CzFAD7B are in the group II of ω3 FADs from Viridiplantae including CrFAD7, AtFAD7 and AtFAD8 that are localized in the plastid and introduce a double bond on the *n*-3 positions of C18 and C16 PUFAs [21, 22, 24]; CzFAD5A, CzFAD5B and CzFAD5C are in the group VI of ∆7 FADs with AtFAD5 that desaturates C16:0 in the *sn*-2 position of MGDG and probably digalactosyldiacylglycerol (DGDG) [14, 15]; CzFAD3A and CzFAD3B fall in the group VII of ∆4/∆5/∆6 FADs (front-end FADs) and are closely related to *C. reinhardtii* FAD3 (CrFAD3 or Cr∆4FAD) that introduces a ∆4 double bond on MGDG-linked C16 PUFAs with a preexisting ∆7 double bond [25]; CzFAD4 is in the group IX of ∆3(trans) FADs including AtFAD4 that introduces a *trans* double bond on ∆3 of C16:0 in *sn*-2 position of PG [13].

### Functional validation of *CzFAD* genes in *Saccharomyces cerevisiae*

**Fig. 1:**
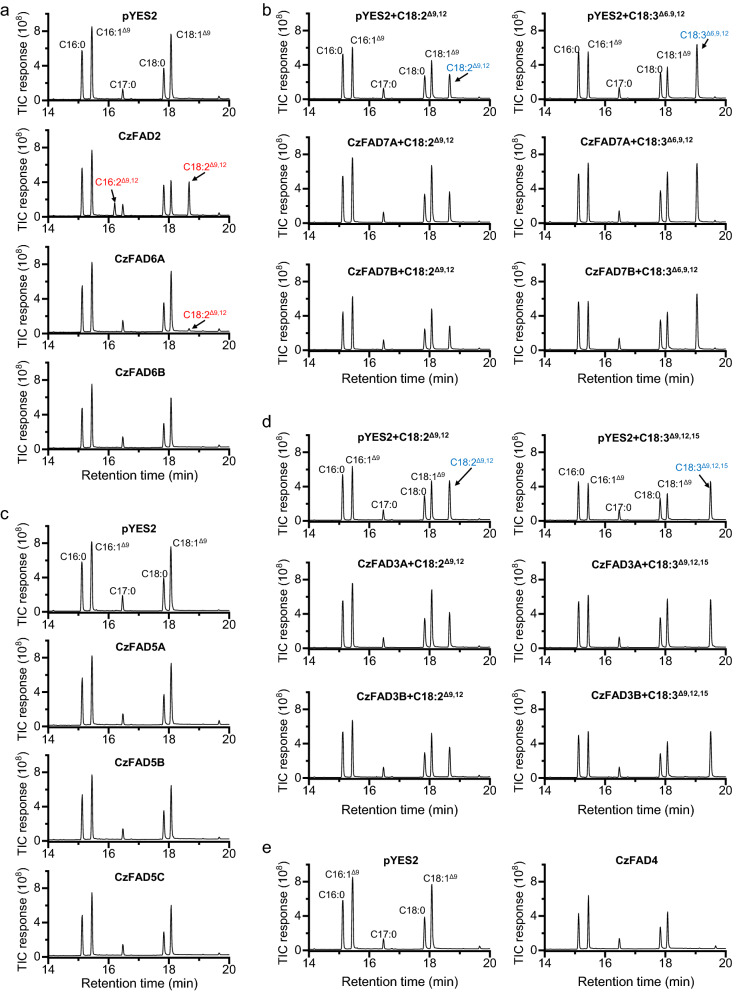
GC–MS chromatography of fatty acids from *S. cerevisiae* expressing the empty vector pYES2 and *CzFAD* genes. **a** *S. cerevisiae* transformant with pYES2, *CzFAD2*, *CzFAD6A* or *CzFAD6B*. **b** *S. cerevisiae* transformant with pYES2, *CzFAD7A* or *CzFAD7B* supplemented with FFA C18:2^Δ9,12^ or C18:3^Δ6,9,12^. **c** *S. cerevisiae* transformant with pYES2, *CzFAD5A*, *CzFAD5B* or *CzFAD5C*. **d** *S. cerevisiae* transformant with pYES2, *CzFAD3A* or *CzFAD3B* supplemented with FFA C18:2^Δ9,12^ or C18:3^Δ9,12,15^. **e** *S. cerevisiae* transformant with pYES2 or *CzFAD4*. Newly synthesized and added fatty acids are designated in red and blue, respectively. C17:0 was added as the internal control

To validate the functionality of *CzFAD* genes, their coding sequences were each sub-cloned into the yeast expression vector pYES2-CT and introduced to the baker yeast *S. cerevisiae* for heterologous expression. The presence of *CzFAD* genes was confirmed by colony PCR (Additional file 1: Figure S7a). *S. cerevisiae* with the empty vector contained four FAs, i.e., C16:0, C16:1^∆9^, C18:0 and C18:1^∆9^ (Fig. 1a). Expression of *CzFAD2* allowed the yeast produce new FAs C18:2^∆9,12^ and C16:2^∆9,12^ (Fig. 1a), demonstrating that CzFAD2 is a ∆12 FAD. Quantification of FA profiles showed that the desaturation efficiencies (product/[product + substrate] × 100) on C18:1^∆9^ and C16:1^∆9^ were 50.0% and 14.8%, respectively (Table 1), indicating that CzFAD2 prefers C18:1^∆9^ as the substrate. Expression of *CzFAD6A* also led to new FA formation, but only C18:2^∆9,12^ in a very low abundance (1.3%) relative to the total FAs (TFA) (Fig. 1a and Table 1).

**Table 1:** Fatty acid composition in *S. cerevisiae* expressing the empty vector pYES2, *CzFAD2*, *CzFAD6A*, or *CzFAD6B*

Fatty acids (%)	pYES2	CzFAD2	CzFAD6A	CzFAD6B
C16:0	21.8 ± 0.8	21.8 ± 1.1	21.8 ± 1.7	22.1 ± 2.0
C16:1^Δ9^	36.3 ± 1.5	31.4 ± 1.5	36.1 ± 0.9	37.2 ± 2.1
C16:2^Δ9,12^	–	5.47 ± 0.52	–	–
C18:0	13.8 ± 0.9	13.9 ± 1.2	13.5 ± 1.5	14.1 ± 1.9
C18:1^Δ9^	28.0 ± 1.9	13.7 ± 1.5	27.3 ± 2.1	26.6 ± 1.6
C18:2^Δ9,12^	–	13.7 ± 0.8	1.3 ± 0.1	–

On the other hand, expression of *CzFAD6B*, *CzFAD7A*, *CzFAD7B*, *CzFAD5A*, *CzFAD5B*, *CzFAD5C*, *CzFAD3A*, *CzFAD3B* or *CzFAD4* in yeast did not produce any new FA, even supplemented with the putative FA substrates (Fig. 1b–e). Probably, the FA substrates are esterified to lipids not recognized by the CzFADs as the lipid profiles differ greatly between yeast and the green alga *C. zofingiensis* [48, 57], or certain co-factors required for the desaturation activity of these enzymes are not available within the compartments where the heterologously expressed CzFADs reside in yeast [8, 58]. It is also possible that some of them are not authentic FADs, e.g., CzFAD6B that lacks the conserved histidine boxes (Additional file 1: Figure S4).

### Functional validation of *CzFAD* genes in *Synechococcus elongatus*

It is believed that cytochrome b5 serves as the electron donor for the ER FADs while ferredoxin is used by the plastidial, cyanobacterial and bacterial FADs [8]. In this context, the plastidial FADs such as CzFAD7s may not function in yeast. Therefore, we also introduced *CzFAD* genes (sub-cloned in the cyanobacterial vector pSyn6) to the cyanobacterium *S. elongatus* for functional validation. The presence of *CzFAD* genes was also confirmed by colony PCR (Additional file 1: Figure S7b).

### *CzFAD2 and CzFAD6A but not CzFAD6B showed ∆12 activities on both C16:1*^*∆9*^* and C18:1*^*∆9*^

**Fig. 2:**
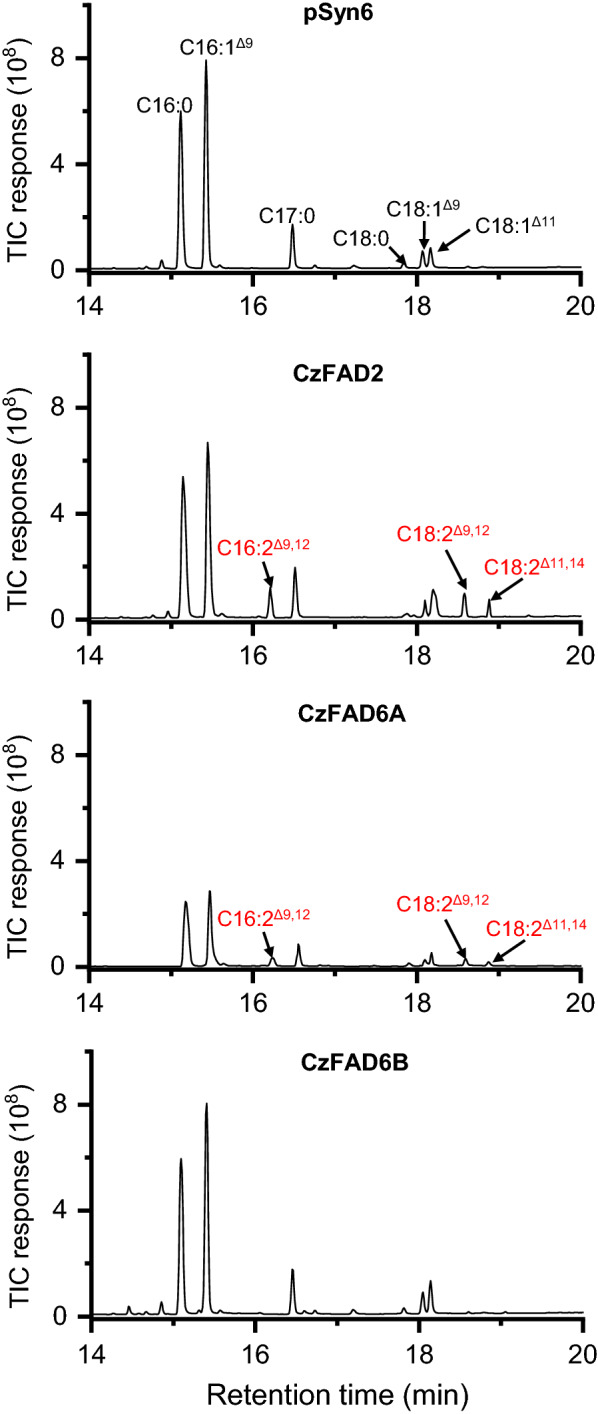
GC–MS chromatography of fatty acids from *S. elongatus* expressing the empty vector pSyn6, *CzFAD2*, *CzFAD6A* or *CzFAD6B.* Newly synthesized fatty acids are designated in red. C17:0 was added as the internal control. The mass spectra of C16:2^Δ9,12^ and C18:2^Δ11,14^ are shown in Additional file 2: Figure S8

*S. elongatus* transformed with the empty vector contained FAs of C16:0, C16:1^∆9^, C18:0, C18:1^∆9^ and C18:1^∆11^ (Fig. 2). Like in *S. cerevisiae* (Fig. 1a), expression of *CzFAD2* in *S. elongatus* led to synthesis of C18:2^∆9,12^ and C16:2^∆9,12^ (Fig. 2) and CzFAD2 had a higher desaturation efficiency on C18:1^∆9^ than on C16:1^∆9^ (36.7% verse 9.0%) based on the quantification of FA profiles (Table 2). Expression of *CzFAD6A* in *S. elongatus* also produced C18:2^∆9,12^ and C16:2^∆9,12^ (Fig. 2) and their relative abundances were higher than that in the *CzFAD2*-expressing *S. elongatus* (Table 2) or *CzFAD6A*-expressing *S. cerevisiae* (Table 1), supporting that *S. elongatus* is more suitable for functional validation of plastidial FADs. CzFAD6A also exhibited a higher desaturation efficiency on C18:1^∆9^ than on C16:1^∆9^ (45.6% verse 16.4%). Expression of *CzFAD6B* in *S. elongatus*, on the other hand, did not produce new FA (Fig. 2 and Table 2), as the case in *S. cerevisiae* (Table 1). The functional failure of CzFAD6B in *S. cerevisiae* and *S*. *elongatus*, together with the fact that CzFAD6B lacks the conserved histidine boxes (Additional file 1: Figure S4) and is less related to CrFAD6 and AtFAD6 than CzFAD6A (Additional file 1: Figure S6), suggests that CzFAD6B is not a genuine ω6 FAD.

**Table 2:** Fatty acid composition in *S. elongatus* expressing the empty vector pSyn6, *CzFAD2, CzFAD6A* or *CzFAD6B* without or with FFA feeding

Fatty acids (%)	Without FFA feeding				With C16:1^Δ9^ feeding				With C18:1^Δ9^ feeding				With C18:1^Δ11^ feeding			
	pSyn6	CzFAD2	CzFAD6A	CzFAD6B	pSyn6	CzFAD2	CzFAD6A	CzFAD6B	pSyn6	CzFAD2	CzFAD6A	CzFAD6B	pSyn6	CzFAD2	CzFAD6A	CzFAD6B
C16:0	44.5 ± 3.4	42.7 ± 2.9	40.7 ± 2.1	44.9 ± 1.1	35.3 ± 1.1	33.5 ± 2.2	30.4 ± 2.5	38.1 ± 2.6	35.2 ± 2.0	35.0 ± 1.7	36.5 ± 1.0	36.2 ± 3.0	35.9 ± 3.0	33.4 ± 2.0	30.7 ± 2.0	36.5 ± 1.9
C16:1^Δ9^	41.6 ± 2.3	34.6 ± 1.9	32.7 ± 1.4	40.3 ± 1.0	46.8 ± 3.2	39.7 ± 2.0	35.3 ± 1.8	44.9 ± 1.8	32.8 ± 1.3	29.8 ± 1.1	28.6 ± 2.9	33.2 ± 1.7	33.6 ± 3.0	27.2 ± 2.2	27.6 ± 2.3	30.9 ± 3.0
C16:2^Δ9,12^	–	3.4 ± 0.4	6.4 ± 1.0	–	–	8.1 ± 0.9	10.5 ± 0.8	–	–	4.0 ± 0.9	6.1 ± 0.3	–	–	3.3 ± 1.0	5.1 ± 1.0	–
C18:0	1.2 ± 0.0	1.3 ± 0.1	1.3 ± 0.3	1.7 ± 0.2	1.7 ± 0.3	1.2 ± 0.1	0.9 ± 0.0	1.4 ± 0.3	1.5 ± 0.0	1.5 ± 0.1	1.1 ± 0.1	1.3 ± 0.3	1.0 ± 0.3	1.1 ± 0.3	1.0 ± 0.0	1.4 ± 0.5
C18:1^Δ9^	4.4 ± 0.5	5.0 ± 1.0	4.8 ± 0.1	4.3 ± 1.0	4.0 ± 1.0	4.0 ± 0.8	4.1 ± 1.2	4.0 ± 0.8	23.7 ± 1.4	12.7 ± 2.6	10.3 ± 1.2	22.5 ± 2.7	3.5 ± 0.8	3.9 ± 0.1	3.8 ± 0.9	3.4 ± 1.0
C18:1^Δ11^	8.3 ± 1.0	8.3 ± 1.3	7.7 ± 0.3	8.7 ± 1.3	12.2 ± 1.0	9.6 ± 1.0	10.8 ± 1.6	11.6 ± 1.0	6.8 ± 0.7	7.5 ± 0.4	7.1 ± 1.1	6.8 ± 1.0	26.1 ± 2.3	27.5 ± 3.1	26.6 ± 1.1	27.9 ± 3.0
C18:2^Δ9,12^	–	2.9 ± 0.4	4.0 ± 0.9	–	–	2.5 ± 0.0	3.4 ± 0.4	–	–	5.9 ± 0.6	8.1 ± 1.3	–	–	2.3 ± 0.7	3.2 ± 0.2	–
C18:2^Δ11,14^	–	1.8 ± 0.1	2.4 ± 0.2	–	–	3.3 ± 0.1	4.6 ± 0.3	–	–	1.7 ± 0.1	2.2 ± 0.2	–	–	1.3 ± 0.0	1.9 ± 0.3	–

Interestingly, expression of *CzFAD2* or *CzFAD6A* in *S. elongatus* led to synthesis of an additional FA, C18:2^∆11,14^ (Fig. 2). It might be derived from the desaturation of C18:1^∆11^ mediated by CzFAD2/CzFAD6 or from elongation of C16:2^∆9,12^ by the action of an endogenous elongase. To confirm the source of C18:2^∆11,14^, we analyzed the FA profiles as affected by supplementation of exogenous free FAs (FFAs) including C16:1^∆9^, C18:1^∆9^ and C18:1^∆11^. When supplied with C16:1^∆9^, higher abundance of C18:1^∆11^ was observed in *S. elongatus* transformed with the empty vector (Table 2), consistent with the previous report [59] and suggesting that C18:1^∆11^ is from the elongation of C16:1^∆9^ rather than the desaturation of C18:0. In *S. elongatus* transformed with either *CzFAD2* or *CzFAD6*, C16:1^∆9^ supplementation led to increased abundances of C16:2^∆9,12^ (2.4- or 1.6-fold) and its elongated product C18:2^∆11,14^ (1.8- or 1.9-fold), while C18:1^∆11^ addition did not promote C18:2^∆11,14^ abundance (Table 2), supporting that C18:2^∆11,14^ is from C16:2^∆9,12^ elongation (similar to formation of C18:1^∆11^ from C16:1^∆9^) instead of C18:1^∆11^ desaturation. Together with that C18:1^∆9^ addition caused considerably increased abundance of C18:2^∆9,12^ (Table 2), CzFAD2 and CzFAD6A function in introducing a double bond on the ∆12 rather than ∆14 position of C18:1^∆9^, resembling the ER and plastidial ∆12 FADs from *Phaeodactylum tricornutum* [60].

It is worth noting that unlike *C. zofingiensis* that contains C16:1^∆7^ [48], *S. elongatus* has no C16:1^∆7^ but C16:1^∆9^ (Fig. 2). We so far have no idea whether CzFAD6A is functional in desaturating C16:1^∆7^ to C16:2^∆7,10^. Nevertheless, CrFAD6, a closely related homolog of CzFAD6, is involved in catalyzing not only C18:1^∆9^ to C18:2^∆9,12^, but also C16:1^∆7^ to C16:2^∆7,10^ in *C. reinhardtii* [55, 56]. Moreover, the homolog of CzFAD6 in Arabidopsis, AtFAD6, also catalyzes C18:1^∆9^ to C18:2^∆9,12^ and C16:1^∆7^ to C16:2^∆7,10^ [18, 19]. In this context, CzFAD6A may resemble CrFAD6 and AtFAD6 and acts on both C18:1^∆9^ and C16:1^∆7^ in the chloroplast of *C. zofingiensis*.

### CzFAD7B rather than CzFAD7A introduced a double bond on the ∆15 position of C18 UFAs and the ∆12 position of C16:1^∆9^

**Fig. 3:**
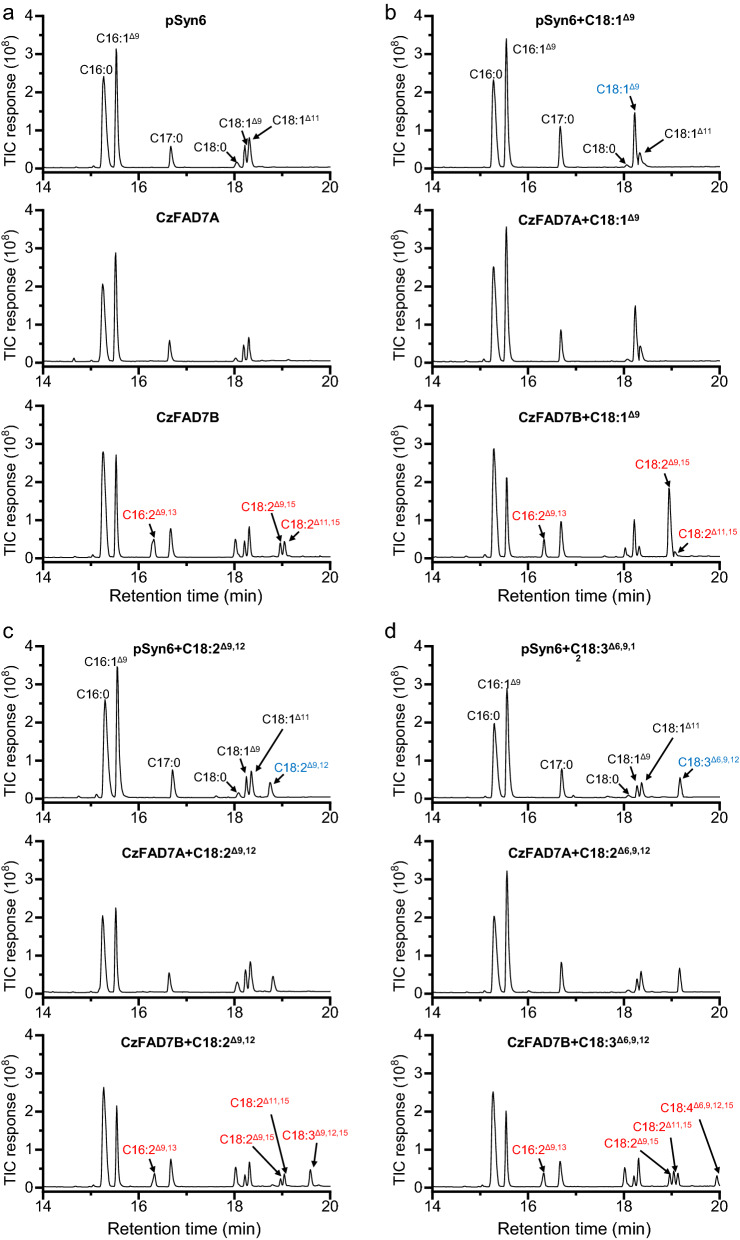
GC–MS chromatography of fatty acids from *S. elongatus* expressing the empty vector pSyn6, *CzFAD7A* or *CzFAD7B* without or with FFA feeding*.* **a** Without FFA feeding. **b** With C18:1^Δ9^ feeding. **c** With C18:2^Δ9,12^ feeding. **d** With C18:3^Δ6,9,12^ feeding. Newly synthesized and added fatty acids are designated in red and blue, respectively. The mass spectra of C16:2^Δ9,13^, C18:2^Δ9,15^ and C18:2Δ^11,15^ are shown in Additional file 2: Figure S8

Phylogenic analysis indicated that CzFAD7A and CzFAD7B belong to ω3 FADs (Additional file 1: Figure S6). To validate their functions, C18 UFAs were added to the *S. elongatus* cultures transformed with the empty vector, *CzFAD7A* or *CzFAD7B*. Like the empty vector pSyn6, expression of *CzFAD7A* failed to give rise to new FAs either without or with feeding of C18:1^∆9^, C18:2^∆9,12^ and C18:3^∆6,9,12^ (Fig. 3), indicative of its null function, at least in *S. elongatus*. By contrast, *CzFAD7B* was demonstrated active in *S. elongatus* even without FFA feeding, leading to formation of three new FAs, namely C16:2^∆9,12^ (6.8% of TFA), C18:2^∆9,15^ (2.8%) and C18:2^∆11,14^ (3.6%) (Fig. 3a and Table 3). C18:2^∆11,14^, as mentioned above, is likely from elongation of C16:2^∆9,12^ rather than desaturation of C18:1^∆11^. In this context, CzFAD7B can introduce a double bond on the ∆12 and ∆15 positions of position of C16:1^∆9^ and C18:1^∆9^, respectively. Supplementation of exogenous C18:1^∆9^ caused a considerable increase in C18:2^∆9,15^ abundance reaching 20.5% of TFA (Fig. 3b and Table 3), further confirming that C18:2^∆9,15^ is desaturated from C18:1^∆9^. When fed with C18:2^∆9,12^, *S. elongatus* expressing *CzFAD7B* produced an additional new FA, C18:3^∆9,12,15^ (Fig. 3c), which accounted for 5.0% of TFA (Table 3). The feeding of C18:3^∆6,9,12^, on the other hand, led to formation of C18:4^∆6,9,12,15^ (Fig. 3d), which accounted for 2.8% of TFA (Table 3). The desaturation efficiencies of CzFAD7B on C18:2^∆9,12^ and C18:3^∆6,9,12^ were 92.5% and 49.4%, respectively, indicating that the desaturase prefers C18:2^∆9,12^ over C18:3^∆6,9,12^ as the substrate.

**Table 3:** Fatty acid composition in *S. elongatus* expressing the empty vector pSyn6, *CzFAD7A* or *CzFAD7B* without or with FFA feeding

Fatty acids (%)	Without FFA feeding			With C18:1^Δ9^ feeding			With C18:2^Δ9,12^ feeding			With C18:3^Δ6,9,12^ feeding		
	pSyn6	CzFAD7A	CzFAD7B	pSyn6	CzFAD7A	CzFAD7B	pSyn6	CzFAD7A	CzFAD7B	pSyn6	CzFAD7A	CzFAD7B
C16:0	44.1 ± 1.6	48.5 ± 2.3	48.1 ± 3.0	40.9 ± 2.0	42.7 ± 1.0	44.8 ± 1.2	44.1 ± 2.1	41.3 ± 2.3	49.6 ± 3.1	41.5 ± 1.7	40.3 ± 2.2	47.9 ± 2.1
C16:1^Δ9^	34.8 ± 2.4	41.8 ± 2.0	24.4 ± 1.2	39.0 ± 1.1	38.1 ± 2.2	18.7 ± 0.9	38.5 ± 2.2	27.0 ± 1.1	21.6 ± 1.6	44.1 ± 3.1	42.1 ± 3.7	20.3 ± 2.5
C16:2^Δ9,13^	–	–	6.8 ± 1.0	–	–	4.4 ± 0.7	–	–	4.7 ± 0.6	–	–	4.6 ± 0.7
C18:0	2.1 ± 0.3	1.2 ± 0.2	4.9 ± 0.6	0.7 ± 0.0	0.6 ± 0.0	2.0 ± 0.1	1.9 ± 0.3	4.6 ± 0.9	5.8 ± 0.6	0.8 ± 0.0	0.9 ± 0.1	5.9 ± 1.1
C18:1^Δ9^	7.5 ± 0.5	4.2 ± 0. 7	2.8 ± 0.3	13.5 ± 1.3	13.8 ± 1.2	7.3 ± 1.0	3.6 ± 0.2	5.2 ± 0.4	2.4 ± 0.4	2.9 ± 0.4	3.2 ± 1.0	2.3 ± 0.3
C18:1^Δ11^	11.5 ± 1.0	4.2 ± 0.8	6.6 ± 1.0	5.9 ± 1.0	4.8 ± 0.7	2.1 ± 0.1	6.8 ± 0.9	15.7 ± 1.4	6.0 ± 0.8	4.6 ± 0.4	6.3 ± 1.2	6.9 ± 0.8
C18:2^Δ9,12^	–	–	–	–	–	–	5.1 ± 0.7	6.2 ± 1.1	0.4 ± 0.0	–	–	–
C18:2^Δ9,15^	–	–	2.8 ± 0.5	–	–	20.5 ± 2.5	–	–	1.5 ± 0.3	–	–	2.8 ± 0.3
C18:2^Δ11,15^	–	–	3.6 ± 0.3	–	–	0.2 ± 0.0	–	–	2.9 ± 0.5	–	–	3.6 ± 1.1
C18:3^Δ6,9,12^	–	–	–	–	–	–	–	–	–	6.1 ± 1.0	6.8 ± 1.2	2.9 ± 0.9
C18:3^Δ9,12,15^	–	–	–	–	–	–	–	–	5.0 ± 0.5	–	–	–
C18:4^Δ6,9,12,15^	–	–	–	–	–	–	–	–	–	–	–	2.8 ± 0.2

Combined, CzFAD7B functions as a ω3/∆12 bi-functional desaturase, with the ω3 (or ∆15) desaturation activity on C18 UFAs being the major and the ∆12 desaturation activity on C16:1^∆9^ being the minor. Several previous studies reported the ω3/∆12 bi-functional desaturases [61–64], but they are mainly derived from fungi. Although CrFAD7, the homolog of CzFAD7B in *C. reinhardtii*, has been functionally characterized [24], whether it can function as a ∆12 desaturase remains unknown. Therefore, CzFAD7B represents the first reported algae-derived ω3/∆12 bi-functional desaturase. Probably, some of ω3 desaturases that arise from the ∆12 desaturase ancestors via independent gene duplication events maintain the ∆12 desaturation activity [61, 62].

### CzFAD5A but not CzFAD5B or CzFAD5C exhibited ∆7 activity on C16:0

**Fig. 4:**
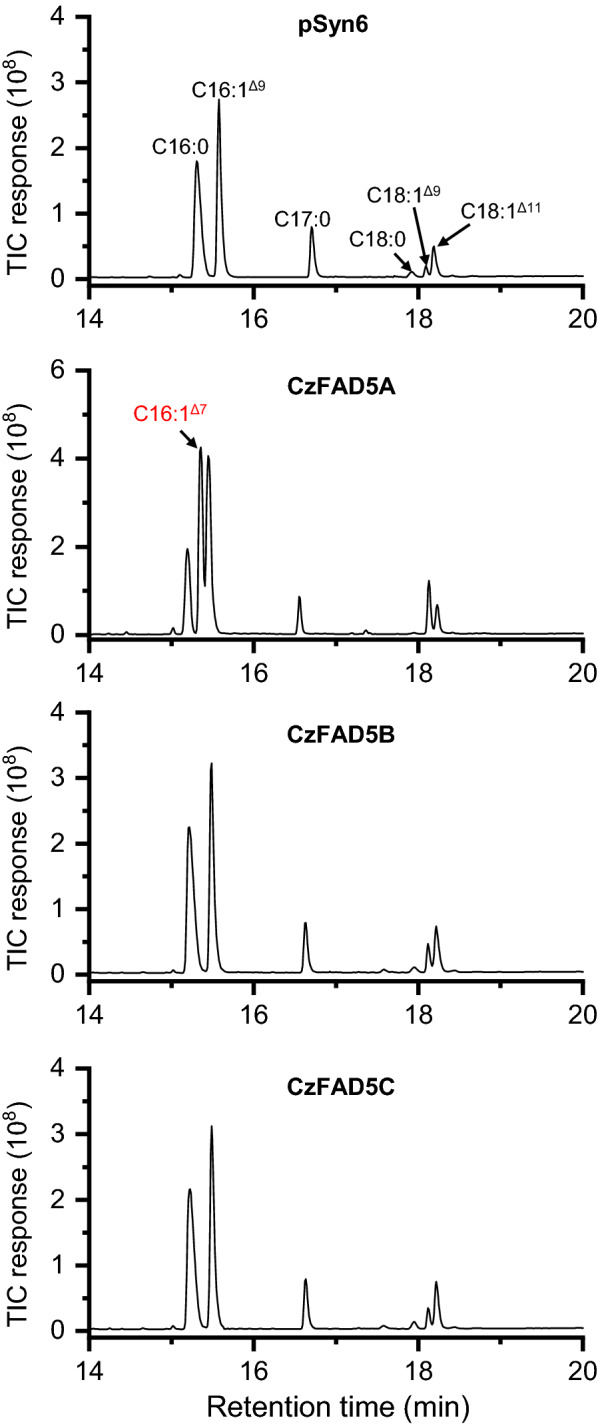
GC–MS chromatography of fatty acids from *S. elongatus* expressing the empty vector pSyn6, *CzFAD5A*, *CzFAD5B* or *CzFAD5C.* Newly synthesized fatty acid is designated in red. The mass spectra of C16:1^Δ7^ are shown in Additional file 2: Figure S8

Compared to the control, the *S. elongatus* transformant carrying *CzFAD5A* contained the newly synthesized C16:1^∆7^ (Fig. 4), which represented 33.5% of TFA and was accompanied by a severe decrease in the abundance of C16:0 (Table 4). Interestingly, the *CzFAD5A-*expressing transformant had a considerably higher abundance of C18:1^∆9^, which reached 8.6% of TFA and was 3.1-fold greater than that in the control (Table 4). C18:1^∆9^ can be derived from the desaturation of C18:0 and/or the elongation of C16:1^∆7^. Without further evidence, it is hard to tell if CzFAD5A possesses a ∆9 desaturation activity and converts C18:0 to C18:1^∆9^. Expression of *CzFAD5B* or *CzFAD5C*, on the other hand, neither produced new FAs nor impacted the FA composition obviously (Fig. 4 and Table 4), suggesting their null function in *S. elongatus*. In this context, CzFAD5A but not CzFAD5B or CzFAD5C is a ∆7 desaturase. Likely, CzFAD5A resembles its homolog in Arabidopsis and functions in desaturating C16:0 to C16:1^∆7^ in the *sn*-2 position of MGDG. The homolog of CzFAD5A in *C. reinhardtii* (Additional file 1: Figure S6), CrFAD5, which remains to be characterized, may possess the similar desaturation function.

**Table 4:** Fatty acid composition in *S. elongatus* expressing the empty vector pSyn6, *CzFAD5A*, *CzFAD5B* or *CzFAD5C*

Fatty acids (%)	pSyn6	CzFAD5A	CzFAD5B	CzFAD5C
C16:0	45.2 ± 1.8	18.2 ± 2.6	45.1 ± 2.9	46.0 ± 3.2
C16:1^Δ7^	–	33.5 ± 4.1	–	–
C16:1^Δ9^	44.5 ± 2.6	34.5 ± 3.2	42.3 ± 2.7	42.4 ± 2.2
C18:0	1.4 ± 0.2	0.1 ± 0.0	1.2 ± 0.2	1.4 ± 0.1
C18:1^Δ9^	2.8 ± 0.4	8.6 ± 0.7	3.9 ± 0.8	2.9 ± 0.3
C18:1^Δ11^	6.1 ± 0.8	5.2 ± 1.1	7.5 ± 1.1	7.4 ± 0.6

### CzFAD3A and CzFAD3B had ∆6 activities on C18 UFAs

**Fig. 5:**
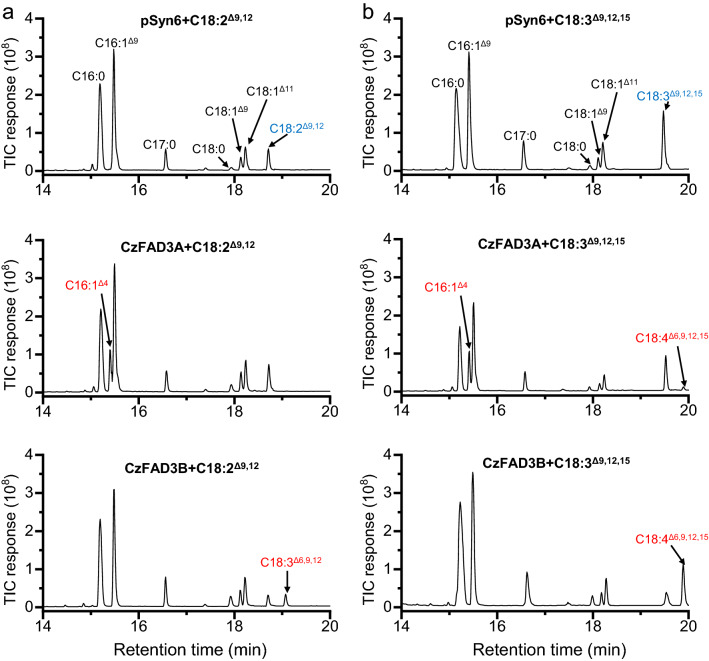
GC–MS chromatography of fatty acids from *S. elongatus* expressing the empty vector pSyn6, *CzFAD3A* or *CzFAD3B* with FFA feeding*.* **a** With C18:2^Δ9,12^ feeding. **b** With C18:3^Δ9,12,15^ feeding. Newly synthesized and added fatty acids are designated in red and blue, respectively. The mass spectra of C16:1^Δ4^ are shown in Additional file 2: Figure S8

The expression of *CzFAD3A* in *S. elongatus*, when supplemented with exogenous C18:2^∆9,12^, did not produce the desaturated product (Fig. 5a). By contrast, the supplementation of C18:3^∆9,12,15^ led to formation of C18:4^∆6,9,12,15^, though in a trace abundance (Fig. 5b). FA quantification showed that the desaturation efficiency of CzFAD3A on C18:3^∆9,12,15^ was only 6.5% (Table 5). CzFAD3B, on the other hand, had activities on both C18:2^∆9,12^ and C18:3^∆9,12,15^ and caused synthesis of C18:3^∆6,9,12^ and C18:4^∆6,9,12,15^ in *S. elongatus*, respectively (Fig. 5a, b). CzFAD3B exhibited a lower desaturation efficiency on C18:2^∆9,12^ (48.7%) than on C18:3^∆9,12,15^ (75.1%), indicating that the desaturase prefers the latter (ω3 type) over the former (ω6 type) as the substrate.

**Table 5:** Fatty acid composition in *S. elongatus* expressing the empty vector pSyn6, *CzFAD3A* or *CzFAD3B*

Fatty acids (%)	Without FFA feeding			With C18:2^Δ9,12^ feeding			With C18:3^Δ9,12,15^ feeding		
	pSyn6	CzFAD3A	CzFAD3B	pSyn6	CzFAD3A	CzFAD3B	pSyn6	CzFAD3A	CzFAD3B
C16:0	40.0 ± 3.2	38.1 ± 2.7	36.7 ± 2.2	39.9 ± 2.3	31.7 ± 1.9	38.9 ± 4.0	38.9 ± 3.6	30.7 ± 1.1	46.1 ± 1.5
C16:1^Δ4^	–	12.7 ± 1.1	–	–	8.7 ± 1.2	–	–	12.3 ± 1.1	–
C16:1^Δ9^	45.1 ± 5.1	40.6 ± 3.1	43.8 ± 1.9	42.7 ± 5.0	39.1 ± 1.4	37.1 ± 2.7	35.1 ± 1.4	35.0 ± 2.0	34.8 ± 3.2
C18:0	1.1 ± 0.2	1.6 ± 0.1	2.8 ± 0.2	1.1 ± 0.2	2.1 ± 0.3	3.7 ± 0.2	1.2 ± 0.1	1.4 ± 0.6	1.5 ± 0.1
C18:1^Δ9^	4.1 ± 0.9	2.2 ± 0.3	5.2 ± 0.9	3.1 ± 0.5	4.3 ± 0.7	4.2 ± 0.1	2.4 ± 0.3	2.2 ± 0.2	1.7 ± 0.1
C18:1^Δ11^	8.7 ± 1.8	4.8 ± 0.7	11.6 ± 1.0	6.7 ± 0.7	7.9 ± 0.6	9.3 ± 1.6	7.1 ± 0.9	5.0 ± 1.4	4.7 ± 0.3
C18:2^Δ9,12^	–	–	–	6.6 ± 0.2	6.2 ± 1.4	3.5 ± 0.3	–	–	–
C18:3^Δ6,9,12^	–	–	–	–	–	3.4 ± 0.6	–	–	–
C18:3^Δ9,12,15^	–	–	–	–	–	–	15.4 ± 1.1	12.6 ± 1.3	2.9 ± 0.2
C18:4^Δ6,9,12,15^	–	–	–	–	–	–	–	0.9 ± 0.0	8.3 ± 0.7

Interestingly, the expression of *CzFAD3A* but not *CzFAD3B* in *S. elongatus* led to formation of C16:1^∆4^, regardless of FFA feeding or not (Additional file 1: Figure S9 and Fig. 5), suggesting that CzFAD3A possesses ∆4 activity. The close homolog of CzFAD3A in *C. reinhardtii*, CrFAD3 (also named as Cr∆4FAD), also possesses ∆4 activity yet on C16:3^∆7,10,13^ to form C16:4^∆4,7,10,13^ [25]. Considering that *C. zofingiensis* consists of similar C16 UFAs as *C. reinhardtii* [48], CzFAD3A may have activity on C16:3^∆7,10,13^ as well. Unfortunately, this FFA is not commercially available and thus was not examined in the present study. It is worth noting that *C. reinhardtii* lacks the ∆6 UFAs C18:3^∆6,9,12^ and C18:4^∆6,9,12,15^ [23, 24], indicating that CrFAD3 has no ∆6 activity [25].

### CzFAD4 acted on C16:0 by adding a ∆3 trans double bond

**Fig. 6:**
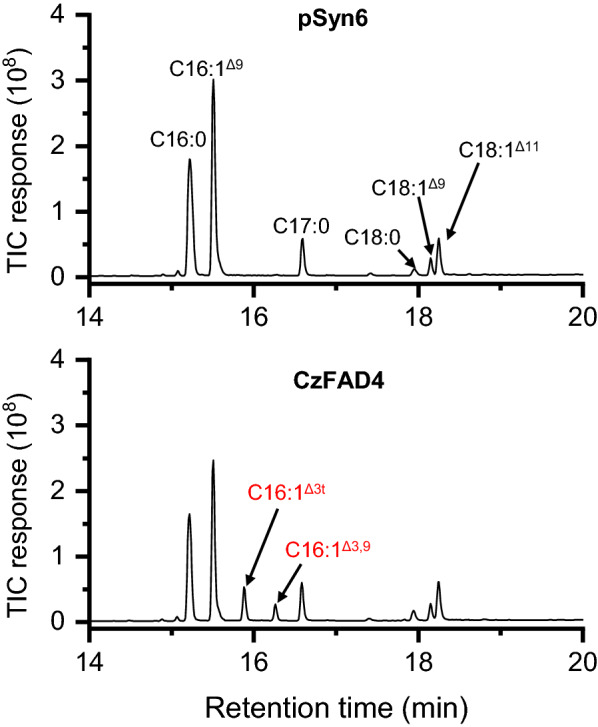
GC–MS chromatography of fatty acids from *S. elongatus* expressing the empty vector pSyn6 or *CzFAD4*. Newly synthesized fatty acids are designated in red. The mass spectra of C16:1^Δ3t^ and C16:1^Δ3,9^ are shown in Additional file 2: Figure S8

Compared to the control, *S. elongatus* expressing *CzFAD4* produced a new FA, C16:1^∆3t^ (a *trans* double bond on the ∆3 position), at the expense of C16:0 (Fig. 6 and Table 6), confirming that CzFAD4 possesses ∆3t activity on C16:0, as is the case for AtFAD4 [13]. The transformant contained an additional new peak right after C16:1^∆3t^ (Fig. 6). Its mass spectrometry data are similar to C16:2^∆3,9^ produced by heterologous expression of *AtFAD4* and an associated redox protein in yeast [58]. In this context, CzFAD4 likely resemble its homolog AtFAD4 and can introduce a ∆3 *trans* double bond on C16:0 and a ∆3 double bond on C16:1^∆9^. As Arabidopsis, *C. zofingiensis*, and *C. reinhardtii* all lack C16:1^∆9^, C16:2^∆3,9^ is not found in the these organisms. These results provide clues for the function of CrFAD5, which is highly related to CzFAD5A (Additional file 1: Figure S6) and awaiting characterization.

**Table 6:** Fatty acid composition in *S. elongatus* expressing the empty vector pSyn6 or *CzFAD4*

Fatty acids (%)	pSyn6	CzFAD4
C16:0	38.9 ± 3.0	32.8 ± 2.7
C16:1^Δ9^	48.3 ± 2.6	39.9 ± 1.8
C16:1^Δ3t^	–	7.7 ± 1.2
C16:2^Δ3,9^	–	3.7 ± 0.7
C18:0	1.7 ± 0.3	2.7 ± 0.3
C18:1^Δ9^	3.1 ± 0.4	3.5 ± 0.7
C18:1^Δ11^	8.1 ± 0.9	9.6 ± 0.8

### Transcriptional expression of *CzFAD* genes in *C. zofingiensis* upon stress conditions

**Fig. 7:**
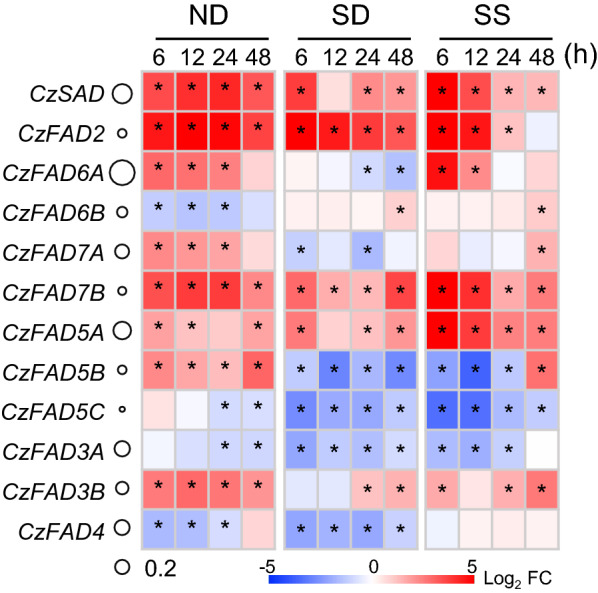
Transcriptional level of *CzFAD* genes in *C. zofingiensis* determined by RT-qPCR. A heat map was used to show the log_2_ transformed fold change (FC) of the transcript level relative to 0 h. The circle right before the heat map designates the transcript abundance of *CzFAD* genes (0 h) relative to the internal control β-actin gene. Data are expressed as mean ± standard deviation (n = 3). An asterisk indicates significant difference when log_2_FC > 1 or < -1 and p < 0.01 (Student’s *t*-test)

*C. zofingiensis* reproduces itself under favorable growth conditions and tends to accumulate triacylglycerol (TAG) under stress conditions such as nitrogen deprivation (ND), sulfur deprivation (SD) and salt stress (SS), etc. [28, 30, 37, 44, 45, 65]. To see how *CzFAD* genes in *C. zofingiensis* respond to these stress conditions at the transcriptional levels, RT-qPCR was performed in a time-resolved manner. The gene expression was presented as the Log_2_ transformed fold changes relative to 0 h (Fig. 7). Upon ND, *CzSAD*, *CzFAD2* and *CzFAD7B* were highly up-regulated (> fivefold increase), *CzFAD6A*, *CzFAD7A*, *CzFAD5A*, *CzFAD5B* and *CzFAD3B* were moderately up-regulated (two- to fourfold increase), while *CzFAD6B*, *CzFAD5C*, *CzFAD3A* and *CzFAD4* were moderately down-regulated. When exposed to SD, the expression patterns of most genes were similar to that under ND conditions with several differences: *CzFAD6A*, *CzFAD7A* and *CzFAD5B* were down-regulated by SD but up-regulated by ND, while *CzFAD6B* was up-regulated by SD but down-regulated by ND. Under SS conditions, the gene expression patterns resembled that under ND conditions except for *CzFAD6B*, *CzFAD5B* and *CzFAD4*: *CzFAD6B* was up-regulated and *CzFAD5B* was down-regulated by SS, contrary to that under ND conditions; *CzFAD4* showed little transcriptional difference upon SS but was down-regulated by ND. Taken the three stress conditions for comparison, *CzSAD*, *CzFAD2*, *CzFAD7B*, *CzFAD5A* and *CzFAD3B* were up-regulated, while *CzFAD5C* and *CzFAD3A* were down-regulated under all the three conditions.

### Variation of FAs in *C. zofingiensis* upon stress conditions

**Fig. 8:**
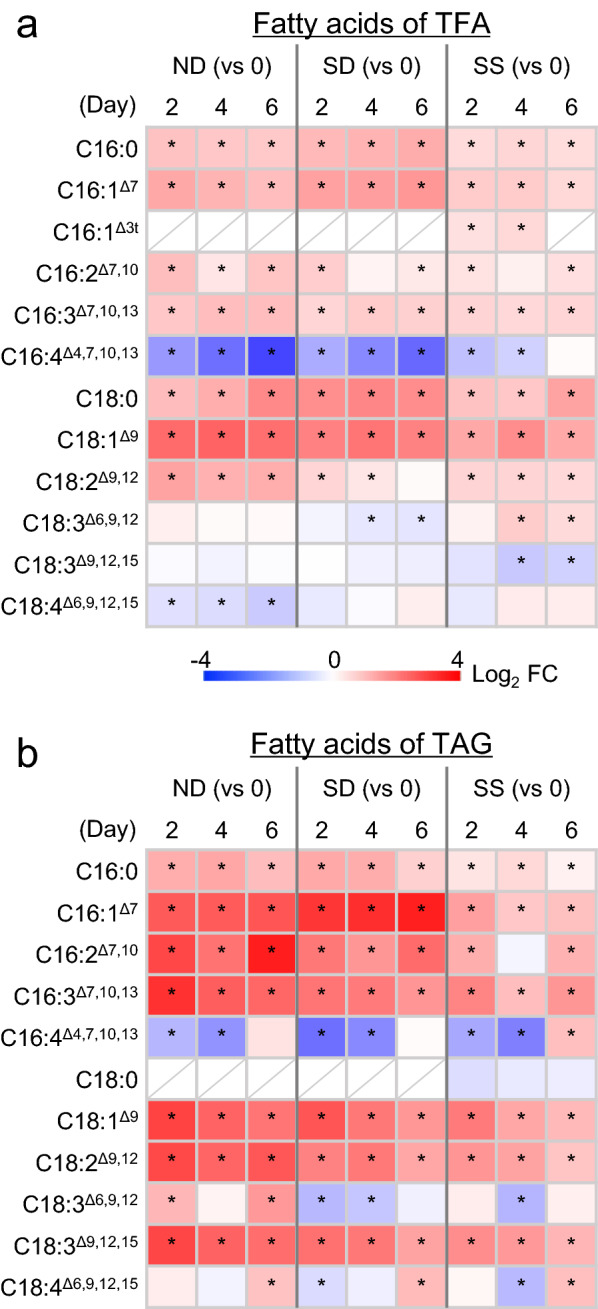
Changes of fatty acids from TFA (a) and TAG (b) of *C. zofingiensis* as affected by various stress conditions of ND, SD and SS. A heat map was used to show the log_2_ transformed fold change (FC) of the fatty acid contents (per dry weight) relative to control (day 0). Data are expressed as mean ± standard deviation (n = 3). An asterisk indicates significant difference when p < 0.01 (Student’s *t*-test)

To understand the changes of FAs in *C. zofingiensis* under different stress conditions, quantification of FAs from both TFA and TAG was carried out. For TFA, the FAs consist of C16:0, C16:1^∆7^, C16:1^∆3t^, C16:2^∆7,10^, C16:3^∆7,10,13^, C16:4^∆4,7,10,13^, C18:0, C18:1^∆9^, C18:2^∆9,12^, C18:3^∆6,9,12^, C18:3^∆9,12,15^ and C18:4^∆6,9,12,15^, with C16:0, C18:1^∆9^, C18:2^∆9,12^ and C18:3^∆9,12,15^ being the major ones (Additional file 2: Table S6). The contents of many FAs showed similar changing patterns in response to the three stress conditions though the extents varied, e.g., C16:0, C16:1^∆7^, C16:2^∆7,10^, C16:3^∆7,10,13^, C18:0, C18:1^∆9^ and C18:2^∆9,12^ increased while C16:4^∆4,7,10,13^ decreased (Fig. 8a). C18:3^∆6,9,12^, C18:3^∆9,12,15^ and C18:4^∆6,9,12,15^, on the other hand, responded differentially to ND, SD and SS (Fig. 8a). C18:1^∆9^ became the most abundant FA under stress conditions, reaching ~ 44.3% of TFA at the expense of the relative abundance of PUFAs such as C18:3^Δ9,12,15^ and C16:4^∆4,7,10,13^ (Additional file 2: Table S6).

The FA composition of TAG was similar to that of TFA, with C16:0, C18:1^∆9^, C18:2^∆9,12^ and C18:3^∆9,12,15^ being the major ones particularly under stress conditions (Additional file 2: Table S7). Most FAs increased in their contents upon all three stress conditions, including C16:0, C16:1^∆7^, C16:2^∆7,10^, C16:3^∆7,10,13^, C18:1^∆9^, C18:2^∆9,12^ and C18:3^∆9,12,15^, and the increase extents were generally greater than that of FAs from TFA (Fig. 8a). C16:4^∆4,7,10,13^, on the other hand, decreased under all three conditions (Fig. 8a). The relative abundance of C18 UFAs showed a considerable increase under stress conditions, ND in particular, which accounted for ~ 72.7% of TFA (Additional file 2: Table S7).

### Profiles of polar membrane lipids and their FA compositions in *C. zofingiensis*

It is believed that the membrane-bound FADs utilize FAs esterified with membrane lipids as substrates for desaturation [8]. Knowledge about the profiles of polar membrane lipids (PMLs) and their FA compositions in *C. zofingiensis* will help understand functional roles of CzFADs. To this end, lipidomic analysis was performed to profile *C. zofingiensis* PMLs by integrating the liquid chromatography–electrospray ionization-mass spectrometry (LC–ESI–MS), thin-layer chromatography (TLC) and gas chromatography–mass spectrometry (GC–MS). LC–ESI–MS analysis of PMLs under favorable growth conditions revealed the lipid species of MGDG, DGDG, sulfoquinovosyl diacylglycerol (SQDG), PG, phosphatidylinositol (PI), phosphatidylethanolamine (PE), phosphatidylcholine (PC) and diacylglycerol-*N*,*N*,*N*-trimethylhomoserine (DGTS) (Additional file 1: Figure S10). Taken together with the FA compositions of each membrane lipid class (Additional file 1: Figure S11) and of *sn*-2 position of each membrane lipid class (Additional file 1: Figure S12), the major species were C18:3^∆9,12,15^/C16:3^∆7,10,13^ and C18:3^∆9,12,15^/C16:4^∆4,7,10,13^ for MGDG, C18:3^∆9,12,15^/C16:3^∆7,10,13^ and C18:3^∆9,12,15^/C18:3^∆9,12,15^ for DGDG, C18:1^∆9^/C16:0, C18:3^∆9,12,15^/C16:0 and C18:2^∆9,12^/C16:0 for SQDG, C18:2^∆9,12^/C16:1^∆3t^, C18:3^∆9,12,15^/C16:1^∆3t^, C18:1^∆9^/C16:1^∆3t^ for PG, C16:0/C18:1^∆9^ and C16:0/C18:2^∆9,12^ for PI, C18:1^∆9^/C18:2^∆9,12^, C18:1^∆9^/C18:3^∆9,12,15^ and C18:1^∆9^/C18:1^∆9^ for PE, C18:1^∆9^/C18:1^∆9^, C18:1^∆9^/C18:2^∆9,12^ and C18:2^∆9,12^/C18:2^∆9,12^ for PC, C16:0/C18:2^∆9,12^, C16:1^∆7^/C18:2^∆9,12^ and C16:0/C18:4^∆6,9,12,15^ for DGTS (Additional file 1: Figure S10).

**Fig. 9:**
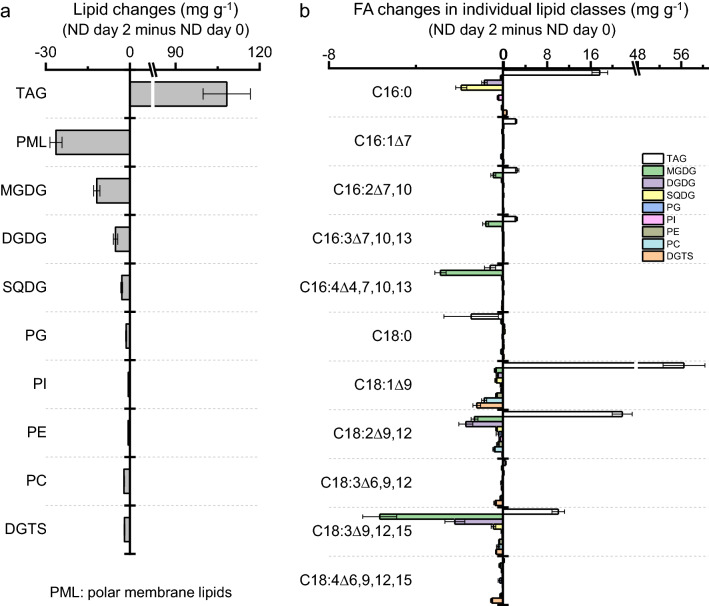
Net lipid changes in *C. zofingiensis* in response to ND. **a** Changes of TAG and polar membrane lipids. **b** Changes of fatty acids in TAG and individual polar membrane lipids. Data, calculated by the values on day 2 of ND minus on day 0 of ND, are expressed as mean ± standard deviation (n = 3)

In response to ND, TAG level showed a considerable increase, accompanied by severe decreases of PMLs particularly the glycolipids such as MGDG, DGDG and SQDG (Fig. 9a). The amount of decreased PMLs, nevertheless, was much smaller than that of increased TAG (i.e., 27 versus 108 mg g^−1^ of dry weight), indicating that turnover of the preexisting PMLs is not enough to support the massive TAG accumulation. When examining the changes of FAs in TAG, the major increased ones were C16:0, C18:1^∆9^, C18:2^∆9,12^ and C18:3^∆9,12,15^, and accounted for 98.1% of total increases; of which, the three C18 UFAs represented 81.8% of total increases (Fig. 9b). C16:0 and C18:1^∆9^ can be de novo synthesized while C18:2^∆9,12^ and C18:3^∆9,12,15^ are derived from the desaturations on PMLs mediated by membrane-bound FADs. MGDG and DGDG contributed to the major amounts of decreased C18:2^∆9,12^ and C18:3^∆9,12,15^ in PMLs (Fig. 9b), likely recycled for TAG assembly. While comparable amounts of C18:3^∆9,12,15^ were observed between decreases in PMLs and increases in TAG, the amount of C18:2^∆9,12^ decrease in PMLs was substantially smaller than that increase in TAG (Fig. 9b). In this context, C18:2^∆9,12^ needs to be synthesized by CzFAD2 and/or CzFAD6A and recycled by unknown enzyme(s) in a continuing manner to meet the requirement of TAG synthesis for C18:2^∆9,12^.

## Discussion

### Reconstruction of FA desaturation pathways in *C. zofingiensis*

Using acetyl-CoA as the precursor and building block, *C. zofingiensis* performs its de novo fatty acid synthesis in the chloroplast [48]. The plastidial acetyl-CoA carboxylase (ACCase), a tetra-subunit enzyme, catalyzes the first committed step of de novo fatty acid synthesis leading to the formation of malonyl-CoA from acetyl-CoA. By the action of malonyl-CoA:ACP transacylase (MCT), malonyl-CoA is converted to malonyl-ACP and then enters the subsequent acyl chain extension cycles. Each cycle adds two carbons to the acyl chain, catalyzed by a series of enzymes including 3-ketoacyl-ACP synthase (KAS), 3-ketoacyl-ACP reductase (KAR), 3-hydroxyacyl-ACP dehydratase (HAD), and enoyl-ACP reductase (ENR). These enzymes are expressed in a coordinated manner leading to the formation of C16:0-ACP and C18:0-ACP [44, 45]. CzSAD, a soluble plastid-localized desaturase, accepts both C16:0-ACP and C18:0-ACP as the substrates yet prefers the latter greatly to produce C18:1^∆9^-ACP [49]. These three acyl-ACPs can be either incorporated into plastidial lipids (e.g., MGDG, DGDG, SQDG and PG) via the ‘prokaryotic pathway’ or released as FFAs by acyl-ACP thioesterase (FAT). Acyls in the plastidial lipids can also be released as FFAs by certain lipases, e.g., plastid galactoglycerolipid degradation1 (PGD1) that has been first characterized in *C. reinhardtii* [66]. These FFAs, assisted with the fatty acid export1 (FAX1) [67, 68], are exported out of the chloroplast and activated by long-chain acyl-CoA synthetase (LACS) [69] and then incorporated into extraplastidial lipids via the ‘eukaryotic pathway’. Both the plastidial and extraplastidial membrane lipids are subjected to membrane-bound FADs for further desaturation.

**Fig. 10:**
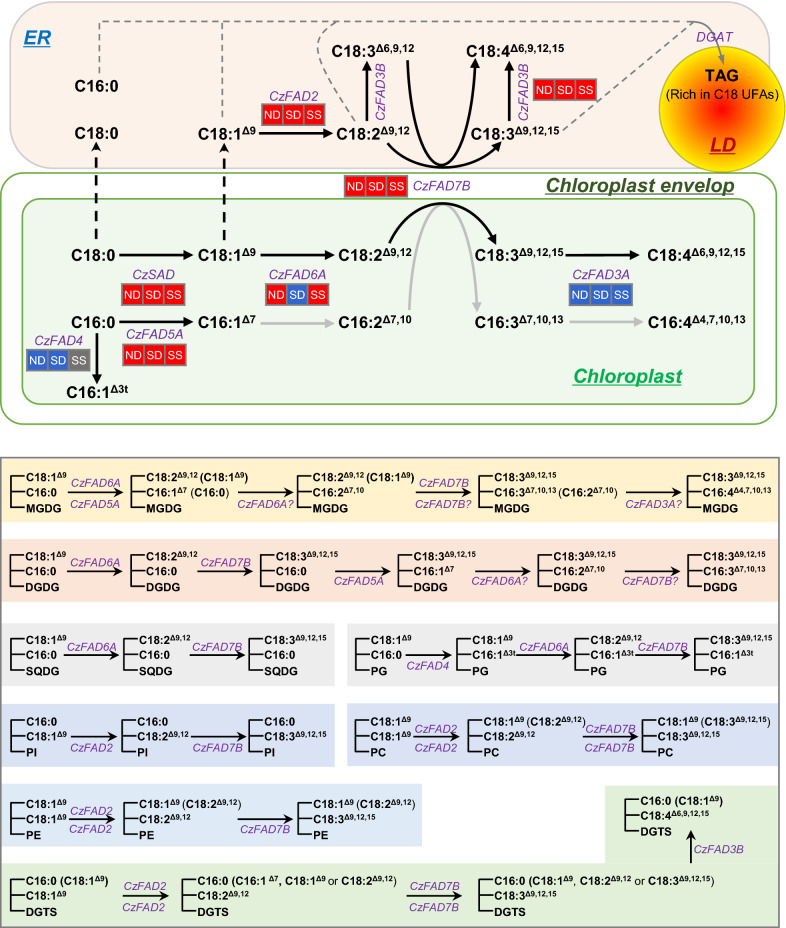
A hypothesized working model illustrating the localization and roles of CzFADs in lipid metabolism in *C. zofingiensis*. ND, nitrogen deprivation; SD, sulfur deprivation; SS, salinity stress. The transcriptional expression pattern of CzFADs under different conditions of ND, SD, and SS is indicated by a background color: red designates up-regulation, blue designates down-regulation while gray indicates non-significant regulation. Gray arrows designate reactions not validated in the present study. CzFAD5B, CzFAD5C, CzFAD6B, CzFAD7A that showed no detected function are not included here. Note that the subcellular localization of FADs is predicted and needs to be experimentally validated. The deduced major desaturation reactions in membrane lipids are depicted in the bottom panel

*C. zofingiensis* genome encodes 11 putative membrane-bound FADs yet only seven of them were demonstrated as active desaturases when expressed in yeast and/or cyanobacterial cells. These include CzFAD2, CzFAD6A, CzFAD7B, CzFAD5A, CzFAD3A, CzFAD3B and CzFAD4 (Figs. 1, 2, 3, 4, 5, 6). Of them, CzFAD2 and CzFAD3B are predicted to be ER-localized while the other five are chloroplast-targeted (Additional file 2: Table S1). Based on the functions and predicted subcellular compartments of these CzFADs and the FA profiles of *C. zofingiensis*, the overall desaturation steps are proposed and depicted in Fig. 10 (top panel). In the chloroplast, C16:0 is converted to C16:1^∆3t^ by CzFAD4 or to C16:1^∆7^ by CzFAD5A. Catalyzed in succession by CzFAD6A, CzFAD7B and CzFAD3A, C16:1^∆7^ is then desaturated to C16:2^∆7,10^, C16:3^∆7,10,13^ and C16:4^∆4,7,10,13^, while C18:1^∆9^ is desaturated to C18:2^∆9,12^, C18:3^∆9,12,15^ and C18:4^∆6,9,12,15^. The desaturation steps in the ER, on the other hand, occur in C18 FAs. C18:1^∆9^ is converted by CzFAD2 to C18:2^∆9,12^, which can be either desaturated first to C18:3^∆6,9,12^ by CzFAD3B then to C18:4^∆6,9,12,15^ by CzFAD7B, or first to C18:3^∆9,12,15^ by CzFAD7B then to C18:4^∆6,9,12,15^ by CzFAD3B.

With respect to each of the membrane lipids, the major desaturation reactions are also deduced and summarized in Fig. 10 (bottom panel). The major MGDG species are C18:3^∆9,12,15^/C16:3^∆7,10,13^ and C18:3^∆9,12,15^/C16:4^∆4,7,10,13^ (Additional file 1: Figure S10). C18:3^∆9,12,15^ in the *sn*-1 position of MGDG is derived from C18:1^∆9^ catalyzed in succession by CzFAD6A and CzFAD7B via the intermediate C18:2^∆9,12^, while C16:4^∆4,7,10,13^ in the *sn*-2 position is from C16:0 catalyzed by CzFAD5A and probably CzFAD6A, CzFAD7B and CzFAD3B via the intermediates C16:1^∆7^, C16:2^∆7,10^ and C16:3^∆7,10,13^. As for DGDG, the major one is C18:3^∆9,12,15^/C16:3^∆7,10,13^ (Additional file 1: Figure S10), which is derived from C18:1^∆9^/C16:0 catalyzed by combinatorial CzFAD6A, CzFAD7B and CzFAD5A. Considering that the *sn*-2 position of SQDG is predominantly occupied by C16:0 (Additional file 1: Figures S10, S12), the desaturation reactions for SQDG are simple and happen in the *sn*-1 position catalyzed by CzFAD6A and CzFAD7B in succession. By contrast, the *sn*-2 position of PG is dominated by C16:1^∆3t^ (Additional file 1: Figures S10, S12), which is produced from C16:0 by the action of CzFAD4. As for the *sn*-1 position of PG, CzFAD6A and CzFAD7B are involved to convert C18:1^∆9^ to C18:3^∆9,12,15^ via the intermediate C18:2^∆9,12^. Of course, it is also possible that certain C18 FAs are desaturated in the extraplastidial membrane lipids and then reimported into the chloroplast for incorporation into the plastidial lipids, which has been indicated in Arabidopsis [70].

Of the four extraplastidial membrane lipids, PI profiles are relatively simple, with C16:0/C18:1^∆9^, C16:0/C18:2^∆9,12^ and C16:0/C18:3^∆9,12,15^ accounting for 91.2% (Additional file 1: Figures S10, S12). The desaturation reactions occur predominantly in the *sn*-2 position of PI catalyzed by CzFAD2 and CzFAD7B. Unlike PI, both PE and PC comprise high-abundance C18 UFAs in their *sn*-1 and *sn*-2 positions (Additional file 1: Figures S10, S12); therefore, desaturation reactions perform in both positions via the involvement of CzFAD2 and CzFAD7B. DGTS is the most abundant extraplastidial membrane lipid in *C. zofingiensis* [48]. The profile of DGTS is complex and consists of 26 species, much greater than other membrane lipids (Additional file 1: Figure S10). Similarly, CzFAD2 and CzFAD7B are involved to catalyze the major desaturation steps in both *sn*-1 and *sn*-2 positions of DGTS. It is worth mentioning that C18:4^∆6,9,12,15^ is present in much higher abundance in DGTS than in other membrane lipids (Additional file 1: Figure S11) and locates predominantly in the *sn*-2 position (Additional file 1: Figures S10, S12). Therefore, the synthesis of C18:4^∆6,9,12,15^, via desaturation of C18:3^∆9,12,15^ mediated by CzFAD3B, occurs mainly in DGTS.

Taken together, likely, CzFAD6A, localized in the chloroplast, functions as an ω6 desaturase with no dependence on substrates such as acyl chains (C16 and C18), lipid head group types (MGDG, DGDG, SQDG and PG) or stereo positions (*sn*-1 and *sn*-2). Similarly, CzFAD2 shows no substrate dependence, but is ER-targeted with access to the extraplastidial membrane lipids. The ω3 desaturase CzFAD7B functions independently on substrates either, yet it likely resides at the plastid outer envelope and can access both plastidial and extraplastidial membrane lipids for desaturation, as proposed for CrFAD7 in *C. reinhardtii* [24]. By contrast, CzFAD5A and CzFAD4 are substrate-specific: the former acts on C16:0 in the *sn*-2 position of MGDG and DGDG, while the latter is restricted to C16:0 in the *sn*-2 position of PG.

### Plastidial and extraplastidial FADs collaborate to determine the abundance of C18 UFAs in *C. zofingiensis*

Under favorable growth conditions, TFA in *C. zofingiensis* is maintained at a relative low and stable level. In response to stress conditions, TFA level tends to increase substantially [37, 45, 65], indicative of a strong stimulation of the de novo FA synthesis. This appears to be universal across TAG-producing algae [71–75]. In support of this, many genes involved in the de novo FA synthesis in *C. zofingiensis* are transcriptionally up-regulated by stress conditions in a considerable and coordinated manner, including ACCase subunit genes, *MCT*, *KAS*, *KAR*, *HAD* and *ENR* [40, 44–46].

When examining the individual FAs of TFA, C16:0, C18:1^∆9^, C18:2^∆9,12^ and C18:3^∆9,12,15^ represented the major ones and had comparable abundances under favorable growth conditions (Additional file 2: Table S6). Upon ND induction, the levels of many FAs increased (Fig. 8a). In agreement with this, CzSAD, CzFAD2, CzFAD6A, CzFAD7B, CzFAD5A and CzFAD3B were transcriptionally up-regulated (Fig. 7). It is worth noting that C18:1^∆9^ exhibited the most increase under ND conditions and its relative abundance (% of TFA) rose from 18.8% to 43.2% on day 2 of ND (Additional file 2: Table S6). Accordingly, CzSAD, the only desaturase so far known that catalyzes the formation of C18:1^∆9^ in *C. zofingiensis*, showed a drastic up-regulation (Fig. 7). C16:1^∆3t^, C16:4^∆4,7,10,13^ and C18:4^∆6,9,12,15^, on the other hand, decreased in response to ND (Fig. 8a). The FADs involved in their formation, e.g., CzFAD4 and CzFAD3A, were down-regulated (Fig. 7). Taking the changing patterns of FAs and transcriptional expression of CzFADs together, FAs in *C. zofingiensis* (content and composition) are controlled by both plastidial and extraplastidial CzFADs at least partly at the transcriptional level. This may partially explain why FAs particularly C18:1^∆9^, C18:2^∆9,12^ and C18:3^∆9,12,15^ had higher levels under ND than under SD or SS (Fig. 8a), because the extent of up-regulation of *CzFAD* genes was generally stronger under ND than that under SD or SS (Fig. 7).

Interestingly, although CzSAD, CzFAD2 and CzFAD7B were up-regulated in comparable degrees in response to ND (Fig. 7), C18:1^∆9^, the product of CzSAD, showed an over twofold increase in the relative abundance, while C18:2^∆9,12^ (product of CzFAD2) exhibited little change and C18:3^∆9,12,15^ (product of CzFAD7B) had an over twofold decrease (Additional file 2: Table S6). It is worth noting that C18:1^∆9^, besides incorporation into membrane lipids for further desaturation, can also be used for TAG assembly mediated by diacylglycerol acyltransferase (DGAT) [76]. In support of this, the type I and type II DGATs from *C. zofingiensis* such as CzDGAT1A and CzDGTT5 accept C18:1^∆9^-CoA and C18:1^∆9^-containing DAGs as substrate for TAG synthesis [77]. As desaturation reactions do not occur in acyls in TAG, the C18:1^∆9^ that is incorporated into TAG for storage would not be further desaturated by CzFAD2 and CzFAD7B, thus leading to enhanced relative abundance of C18:1^∆9^ in *C. zofingiensis*. By contrast, in *C. reinhardtii* under stress conditions such as ND, CrSAD is just stimulated mildly while the desaturases responsible for downstream desaturations of C18:1^∆9^ such as CrFAD2 and CrFAD7 are highly induced [78]. This may partially explain that *C. reinhardtii* has a considerably lower abundance of C18:1^∆9^ yet a higher abundance of C18 PUFAs than *C. zofingiensis*.

### FAs de novo synthesized and recycled from membrane lipids contribute to accumulation of TAG rich in C18 UFAs

In *C. zofingiensis*, TAG is synthesized only at a basal level under favorable growth conditions, with FAs being predominantly esterified with membrane lipids; upon stress conditions such as ND, TAG accumulates substantially and dominates over other lipid classes accompanied by a severe decrease in membrane lipids [28, 44, 48]. This phenomenon is observed not only in green algae, but also in many other oleaginous algae [79–84], indicating that beside de novo FA synthesis, membrane lipid remodeling is involved in contributing acyls to TAG formation.

Our quantitative data of lipids here further support that FAs particularly C18 UFAs recycled from the remodeling of membrane lipids are used for TAG biosynthesis in *C. zofingiensis*. Firstly, when TAG increased in response to ND, we observed a decrease in membrane lipids as a whole and each of the individual membrane lipids as well, though the decrease levels varied greatly depending on the membrane lipid classes (Fig. 9a). Secondly, as shown in Fig. 8b, besides C16:0, C18:0 and C18:1^∆9^, TAG also contained C16 and C18 PUFAs (especially C18:2^∆9,12^ and C18:3^∆9,12,15^) that have to be synthesized in membrane lipids via the action of membrane-bound CzFADs prior to incorporation into TAG. The FAs in membrane lipids can be directly transesterfied to TAG mediated by phospholipid:diacylglycerol acyltransferase (PDAT) or released as FFAs from *sn*-1/2 positions and then incorporated into TAG catalyzed by DGAT [85]. The PDAT from *C. reinhardtii* (CrPDAT) has been characterized and shown to transfer FAs from the *sn*-2 position of a broad range of membrane lipids for TAG synthesis [86]. Nevertheless, it prefers to function under favorable growth conditions and is believed to make a minor contribution to the stress-induced massive TAG synthesis in algae. Similar to *C. reinhardtii*, *C. zofingiensis* harbors a single PDAT [40], which is up-regulated mildly under TAG induction conditions [44, 45]. It may transfer FAs in the *sn*-2 position of membrane lipids (Additional file 1: Figure S12) and contribute to C18 PUFAs in TAG. As for the lipases involved in releasing FAs from membrane lipids, there is so far only one characterized enzyme in microalgae, namely, CrPGD1 from *C. reinhardtii*, which acts on the newly synthesized MGDG to specifically remove C18:1^∆9^ from the *sn*-1 position [66]. *C. zofingiensis* encodes a single PGD1 homolog and it is up-regulated considerably at the transcriptional level under TAG induction conditions [44–46]. If it resembles CrPGD1 and acts specifically on C18:1^∆9^, additional lipase(s) may be needed to release C18:2^∆9,12^ and C18:3^∆9,12,15^ from membrane lipids to support DGAT-mediated incorporation. As a support, the DGAT enzymes in *C. zofingiensis* such as CzDGAT1A and CzDGTT5 have high in vitro activities in using C18:2^∆9,12^-CoA and C18:3^∆9,12,15^-CoA as acyl donors for TAG synthesis [77].

Considering that the PUFAs in TAG have to be derived from membrane lipids and account for 30.9% on day 2 of ND (Additional file 2: Table S7), the remodeling of membrane lipids contributes at least over 30% of TAG synthesis in *C. zofingiensis* under this condition. Moreover, the C16:0, C18:0 and C18:1^∆9^ in TAG may also be recycled from membrane lipids, as is the case for C18:1^∆9^ in *C. reinhardtii* where C18:1^∆9^ from MGDG turnover contributes to ca. 40% of C18:1^∆9^ in TAG [66]. Although it is not easy to quantify contributions of de novo synthesized FAs and membrane lipids-recycled FAs to TAG synthesis, both are believed to participate in *C. zofingiensis*. The *C. zofingiensis* TAG is rich in C18:1^∆9^ (~ 50%) (Additional file 2: Table S7), which is beneficial for the quality of biodiesel and can be further improved by genetic engineering. On one hand, C16:0 remains relatively high in its abundance and represents over 20% of FAs in TAG (Additional file 2: Table S7). Overexpression of KAS II that is responsible for elongating C16:0 to C18:0 may allow more acyl flux to C18:1^∆9^. On the other hand, disturbing the desaturation of C18:1^∆9^ by suppression of CzFAD2 and/or CzFAD6A would lead to buildup of more C18:1^∆9^, which can therefore be incorporated into TAG for storage and protection.

## Methods

### Algal strain and culture conditions

*Chromochloris zofingiensis* (ATCC 30412), purchased from the American Type Culture Collection (ATCC, Rockville, MD, USA), was maintained on an agar plate of modified BG-11 medium at 16 °C with dim light in our lab. When necessary, a single colony from the agar plate was inoculated into 10 mL of liquid medium (in a 100-mL flask) and grown aerobically at 25 °C for 6 days with orbital shaking at 150 rpm and continuous illumination of 30 µmol photons m^−2^ s^−1^. The algal cells were then inoculated at 10% (v/v) into glass columns (3.0 cm in diameter) with continuous illumination of 70 µmol photons m^−2^ s^−1^ and aeration of 1.5% CO_2_ enriched air and grown at 25 °C to late exponential phase (4 days), which were used as seed cultures for subsequent experiments.

Three treatments were applied to *C. zofingiensis*, namely, nitrogen deprivation (ND), sulfur deprivation (SD), and salinity stress (SS). Briefly, the seed cells were centrifuged for 5 min at 5000 g, washed with deionized water and re-suspended at an initial cell density of 0.5 g L^−1^ in fresh BG-11 medium without nitrogen (ND; NaNO_3_ was omitted) or sulfur (MgSO_4_ was replaced with MgCl_2_) or containing 200 mM NaCl (SS). All cultures were grown in 250-mL glass columns with the same parameters mentioned above. The samples from 0, 6, 12, 24 and 48 h of treatments were used for RNA extraction and RT-qPCR, while samples from 0, 2, 4 and 6 days were used for lipid extraction and analysis.

### Cloning and bioinformatics analysis of *C. zofingiensis FAD* genes

Prior to cloning the full-length coding sequence of *C. zofingiensi*s *FAD* genes, their transcription start sites were determined by 5′ rapid amplification of cDNA ends (RACE)-PCR using the SMARTer RACE 5′ Kit (Clontech, CA, USA) and the 5′ gene-specific primers (Additional file 2: Table S3). The amplified fragments, after purified, were subjected to Sanger sequencing for identifying the 5′ UTR and translation start site of each gene. Then, primer pairs (Additional file 2: Table S2) were used to amplify the full-length coding sequence of each *CzFAD* gene (Additional file 1: Figure S1): the forward primer was designed to locate right upstream the start codon based on the confirmed 5′ UTR sequence in our study, and the reverse primer was designed to locate right downstream of the stop codon based on the gene model from Roth et al. [40]. The full-length coding sequences of *CzFAD* genes were verified by sequencing and deposited in NCBI Genbank with accession numbers listed in Additional file 2: Table S1.

Sequence alignment of FAD proteins from various organisms was conducted using ClustalX2.1 with default parameters (http://www.clustal.org/clustal2/) and the phylogenetic tree was generated under MEGA6 using the neighbor-joining method [87]. Conserved domains and transmembrane helices of CzFAD proteins were predicted by the NCBI Conserved Domains Search (https://www.ncbi.nlm.nih.gov/Structure/cdd/wrpsb.cgi) and TMHMM 2.0 (http://www.cbs.dtu.dk/services/TMHMM/), respectively. Subcellular localization prediction was performed using PredAlgo, a multi-subcellular localization prediction tool dedicated to green algae (http://giavap-genomes.ibpc.fr/predalgo), TargetP (http://www.cbs.dtu.dk/services/TargetP/), and WoLF PSORT (https://wolfpsort.hgc.jp/).

### Functional validation of CzFADs in yeast and cyanobacterial cells

The coding sequences of *CzFAD* genes were each PCR amplified using primers from Additional file 2: Table S2 and sub-cloned into the yeast expression vector pYES2-CT (Invitrogen, Carlsbad, CA, USA), or amplified with primers from Additional file 2: Table S4 and cloned into the cyanobacterial vector pSyn6 (Invitrogen).

For heterologous expression in yeast, *CzFAD* genes were each introduced into the *Saccharomyces cerevisiae* strain INVSc1 (*MATa his3*Δ1 *leu2 trp1-289 ura3-52*) (Invitrogen) and selected on SD-URA (synthetic defined medium with uracil omitted) agar plates containing 1% glucose, according to our previously procedures [88]. The empty vector pYES2-CT was also introduced into INVSc1 and used as the control. To induce heterologous gene expression, the positive yeast transformants were cultured in SD-URA liquid medium containing 1% galactose at 30 °C with orbital shaking of 220 rpm for 2 days. Free fatty acids, when necessary, were supplemented to yeast cultures at a concentration of 250 µM upon galactose induction.

For heterologous expression in cyanobacteria, *CzFAD* genes were each introduced into *Synechococcus elongatus* PCC7942 (ATCC, Rockville, MD, USA) and selected on BG-11 agar plates containing 10 μg mL^−1^ of spectinomycin under continuous illumination of 50 μE m^−2^ s^−1^ and temperature of 30 °C, following the manual of GeneArt™ *Synechococcus* Protein Expression Vector. The empty vector pSyn6 was also introduced into *S. elongatus* and used as the control. The putative colonies were validated by genomic PCR. To test the function of CzFADs in *S. elongatus*, positive transformants were grown in liquid BG-11 medium containing 10 μg mL^−1^ of spectinomycin for 7 days, with orbital shaking of 120 rpm, continuous illumination of 50 μE m^−2^ s^−1^ and temperature of 30 °C. When needed, free fatty acids were fed to the *S. elongatus* cultures at a concentration of 250 µM.

### RNA isolation and RT-qPCR analysis

Total RNA extraction from algae samples and removal of contaminated DNA were performed using the plant RNA extraction kit (TaKaRa, Japan) according to the manufacturer's instructions. After purification, the RNA samples were subjected to NanoDrop 2000c (Thermo Scientific, DE, USA) for concentration determination and electrophoresis for quality checking. The cDNA synthesis and quantitative PCR were conducted on a 7500 Fast Real-Time PCR System (Applied Biosystems, Waltham, MA, USA) with SYBR® Premix Ex Taq™ II (Tli RNase H Plus) (TaKaRa), as described previously [44]. Primers used for RT-qPCR are listed in Additional file 2: Table S5. The transcriptional expression levels of *CzFAD* genes were calculated relative to the internal control gene *β-actin*.

### Lipid extraction and analysis

Total lipids were extracted from dried cell samples (yeast, cyanobacterial or algal cells) using a solvent mixture of chloroform/methanol/0.75% NaCl solution (2:1:0.75, by volume), according to our previous methods [44]. The chloroform layer that contains lipids were evaporated under nitrogen gas stream and stored at − 80 °C for later uses.

Thin-layer chromatography (TLC) was used to separate lipids extracted from *C. zofingiensis* samples: neutral lipids were developed on a silica gel TLC plate (Merck, Whitehouse Station, NJ, USA) with a mobile phase of hexane/tert-butyl methyl ether/acetic acid (80/20/2, by volume) and polar lipids were developed with a mobile phase of chloroform/methanol/acetic acid/water (25/4/0.7/0.3, by volume) [44]. The spots of TAG and individual polar membrane lipids on TLC plates were visualized with iodine vapor and recovered. To investigate the FA profile of *sn*-2 position of membrane lipids, each of the recovered membrane lipids was treated with *Rhizopus arrhizus* lipase (Sigma-Aldrich, MO, USA) and the resultant lyso-membrane lipids were recovered again by TLC separation [89].

Total lipids and lipids recovered from TLC plates were transesterified with sulfuric acid in methanol. The resulting fatty acid methyl esters (FAMEs), after extraction from the reaction mixture, were analyzed by using an Agilent 7890 capillary gas chromatograph (GC) equipped with a 5975 C mass spectrometry (MS) detector and a HP-88 capillary column (60 m × 0.25 mm; Agilent Technologies, Wilmington, DE, USA) for quantification [44]. Individual FAMEs were identified and quantified by chromatographic comparison with authentic standards (Sigma, St Louis, MO, USA). C17:0 (Sigma) was used as the internal standard.

The quantification of polar membrane lipids by liquid chromatography–mass spectrometry (LC–MS) was preformed according to the previously described methods [90, 91]. Briefly, the lipids from *C. zofingiensis* samples were dissolved chloroform/methanol (1:1, v/v) and then subjected to analyses on a triple quadrupole MS/MS (Xevo TQ-S, Waters, USA) with electrospray ionization (ESI) source coupled with an Acquity Ultra-Performance Liquid Chromatography (UPLC) system (Waters). MGDG, DGDG, DGTS and PC were analyzed in the positive mode, while PG, SQDG, PE and PI were in the negative mode. Multiple reaction monitoring (MRM) was used for quantitative analysis. The internal standards included MGDG C18:0/C18:0, DGDG C18:0/C18:0, DGTS C16:0/C16:0 d9, PE C17:0/C14:1, PG C17:0/C20:4 and PI C17:0/C20:4. The external standards used for calibration, on the other hand, included MGDG (C16:3/C18:3, C16:3/C18:2 and C16:1/C18:3), DGDG (C16:3/C18:3, C16:3/C18:2 and C16:0/C18:3), DGTS (C16:0/C16:0), PG (C16:0/C18:1 and C18:0/C18:1), PE (C18:0/C18:1), PI (C18:1/C18:1) and SQDG (C16:0/C18:3). The DGTS standard was used for PC quantification as well. Except that MGDG (C18:0/C18:0) and DGDG (C18:0/C18:0) were purchased from Matreya, LLC (Pleasant Gap, PA, USA), other lipid standards were from Avanti Polar Lipids (Alabaster, AL, USA).

### Accession numbers

The full-length coding sequences of *CzFAD* genes are deposited in NCBI GenBank with accession numbers as followed: MT323105 (*CzFAD2*), MT323106 (*CzFAD6A*), MT323107 (*CzFAD6B*), MT323108 (*CzFAD6C*), MT323109 (*CzFAD7A*), MT323110 (*CzFAD7B*), MT323111 (*CzFAD5A*), MT323112 (*CzFAD5B*), MT323113 (*CzFAD5C*), MT323114 (*CzFAD3A*), MT323115 (*CzFAD3B*) and MT323116 (*CzFAD4*).

## Supplementary Information

**Additional file 1: Figure S1.** Comparison between the gene models of *CzFAD*s predicted from Roth et al. [40] and ours confirmed by 5′-RACE and sequencing. **Figure S2.** Characterization of 5′ UTR sequence and cloning of full-length CDS of *CzFAD* genes. **Figure S3.** Conserved domains detected in CzFADs by NCBI Conserved Domains Search. **Figure S4.** Sequence logo and alignment of functional motifs of Δ12, ω6 and ω3 FADs (a), Δ7/Δ9 FADs (b), Δ3^*trans*^ FADs (c), and front-end FADs (d). **Figure S5.** Predicated transmembrane domains for CzFADs by TMHMM. **Figure S6.** Cladogram of fatty acid desaturases of difference functions from various organisms. **Figure S7.** PCR characterization of the *S. cerevisiae* transformants (a) and *S. elongatus* transformants (b) harboring individual *CzFAD* genes. **Figure S8.** The mass spectra of unusual fatty acids (in the form of methyl ester) produced in transformed *S. elongatus*.. **Figure S9.** GC–MS chromatography of fatty acids from *S. elongatus* expressing the empty vector pSy6, *CzFAD3A*, or *CzFAD3B.* Newly synthesized fatty acid is designated in red. **Figure S10.** Relative abundance of species of membrane lipid classes in *C. zofingiensis* under favorable growth conditions. **Figure S11.** Fatty acid relative abundance of individual membrane lipid classes in *C. zofingiensis* under favorable growth conditions. **Figure S12.** Fatty acid relative abundance of *sn*-2 position of individual membrane lipid classes in *C. zofingiensis* under favorable growth conditions.

**Additional file 2: Table S1.** Sequence features of *FAD* genes in *C. zofingiensis.* **Table S2.** Primers used for constructing *CzFAD*s-containing yeast expression vectors. **Table S3.** Primers used for 5′ RACE experiments of *CzFAD* genes. **Table S4.** Primers used for constructing *CzFAD*s-containing *S. elongatus* expression vectors. **Table S5.** Primers used for RT-qPCR of *CzFAD* genes. **Table S6.** Fatty acid composition of TFA in *C. zofingiensis* as affected by various stress conditions of ND, SD and SS. **Table S7.** Fatty acid composition of TAG in *C. zofingiensis* as affected by various stress conditions of ND, SD and SS.

## Data Availability

All data generated or analyzed during this study are included in this published article and its additional information files.

## Abbreviations

DGTS: Diacylglycerol-*N*,*N*,*N*-trimethylhomoserine
ER: Endoplasmic reticulum
FAD: Fatty acid desaturase
FFA: Free fatty acid
GC–MS: Gas chromatography–mass spectrometry
MGDG: Monogalactosyldiacylglycerol
LC–ESI–MS: Liquid chromatography–electrospray ionization-mass spectrometry
MUFAs: Monounsaturated fatty acids
ND: Nitrogen deprivation
PC: Phosphatidylcholine
PE: Phosphatidylethanolamine
PI: Phosphatidylinositol
PG: Phosphatidylglycerol
PMLs: Polar membrane lipids
PUFAs: Polyunsaturated fatty acids
SD: Sulfur deprivation
SQDG: Sulfoquinovosyl diacylglycerol
SS: Salt stress
TAG: Triacylglycerol
UFAs: Unsaturated fatty acids
UTR: Untranslated region
