# Supplementary material for: Characterization of fatty acid desaturases reveals stress-induced synthesis of C18 unsaturated fatty acids enriched in triacylglycerol in the oleaginous alga Chromochloris zofingiensis
Source: Biotechnol Biofuels. 2021 Sep 17;14:184. doi: 10.1186/s13068-021-02037-2 (PMC8447527; doi:10.1186/s13068-021-02037-2)
Supplement: Supplementary file 1 — Additional file 1: Figure S1. Comparison between the gene models of CzFADs predicted from Roth et al. [40] and ours confirmed by 5′-RACE and sequencing. Figure S2. Characterization of 5′ UTR sequence and cloning of full-length CDS of CzFAD genes. Figure S3. Conserved domains detected in CzFADs by NCBI Conserved Domains Search. Figure S4. Sequence logo and alignment of functional motifs of Δ12, ω6 and ω3 FADs (a), Δ7/Δ9 FADs (b), Δ3trans FADs (c), and front-end FADs (d). Figure S5. Predicated transmembrane domains for CzFADs by TMHMM. Figure S6. Cladogram of fatty acid desaturases of difference functions from various organisms. Figure S7. PCR characterization of the S. cerevisiae transformants (a) and S. elongatus transformants (b) harboring individual CzFAD genes. Figure S8. The mass spectra of unusual fatty acids (in the form of methyl ester) produced in transformed S. elongatus.. Figure S9. GC–MS chromatography of fatty acids from S. elongatus expressing the empty vector pSy6, CzFAD3A, or CzFAD3B. Newly synthesized fatty acid is designated in red. Figure S10. Relative abundance of species of membrane lipid classes in C. zofingiensis under favorable growth conditions. Figure S11. Fatty acid relative abundance of individual membrane lipid classes in C. zofingiensis under favorable growth conditions. Figure S12. Fatty acid relative abundance of sn-2 position of individual membrane lipid classes in C. zofingiensis under favorable growth conditions. [file 13068_2021_2037_MOESM1_ESM.pdf]

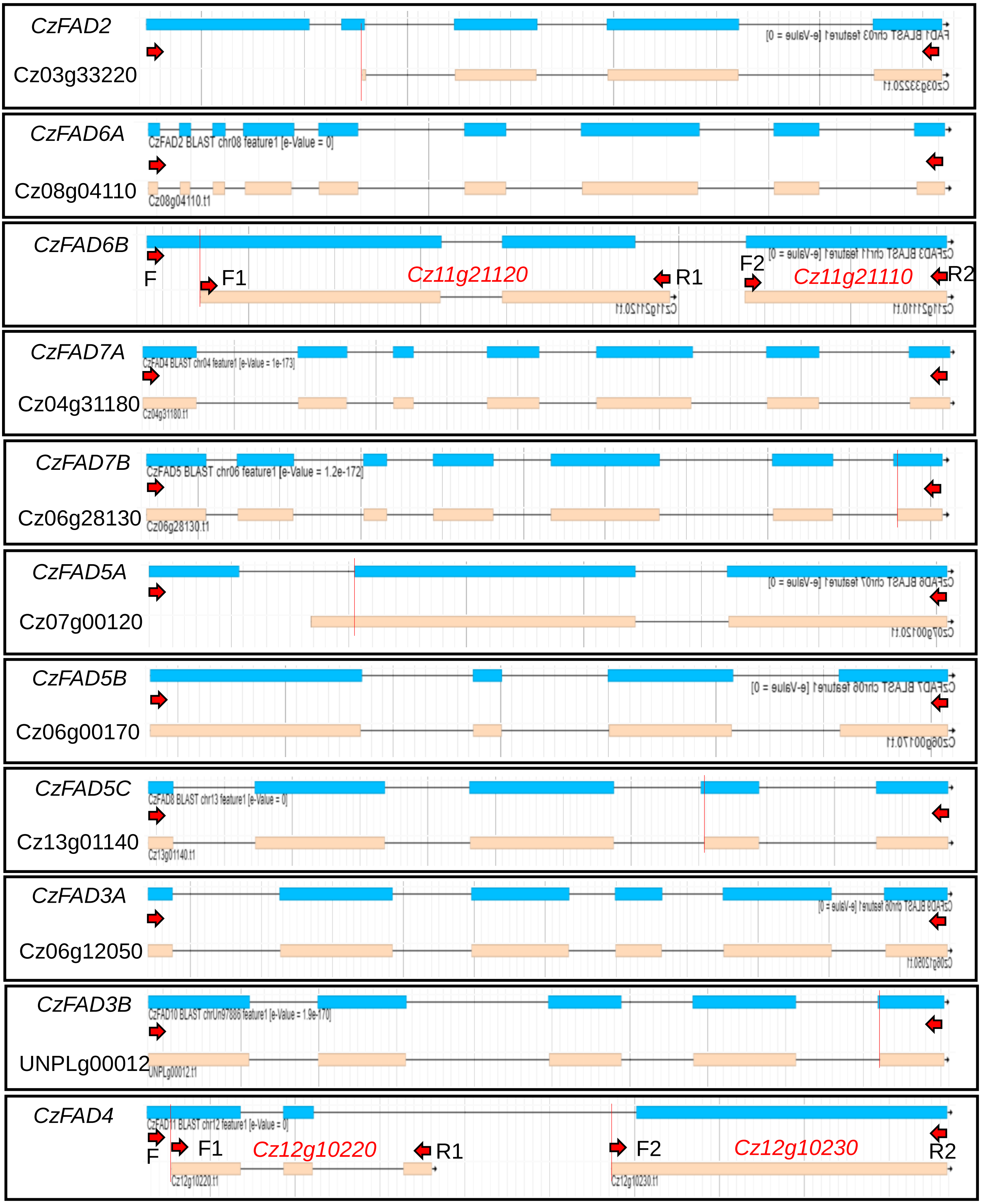

Figure S1. Comparison between the gene models of *CzFAD*s predicted from Roth et al (2017) and ours confirmed by 5′-RACE and sequencing. The gene models from Roth et al. (2017) and us are on the bottom and top of each panel, respectively. The red arrows indicate the primers (listed in Supplemental Table S2) used for cloning the full-length coding sequence.

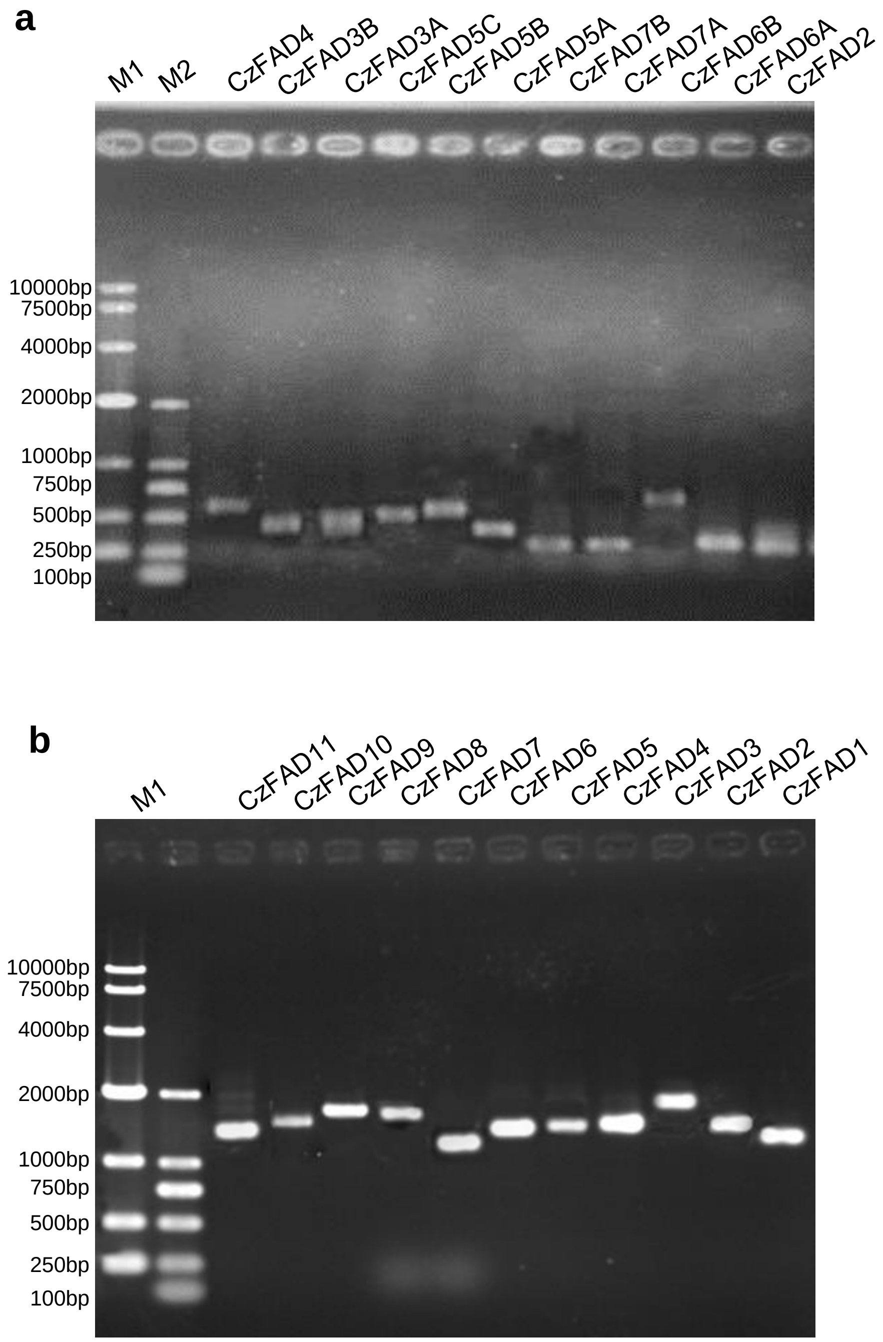

Figure S2. Characterization of 5′ UTR sequence and cloning of full-length CDS of *CzFAD* genes. (a) PCR products of 5′ RACE of *CzFAD* genes. (b) PCR products of full-length CDS of *CzFAD* genes.

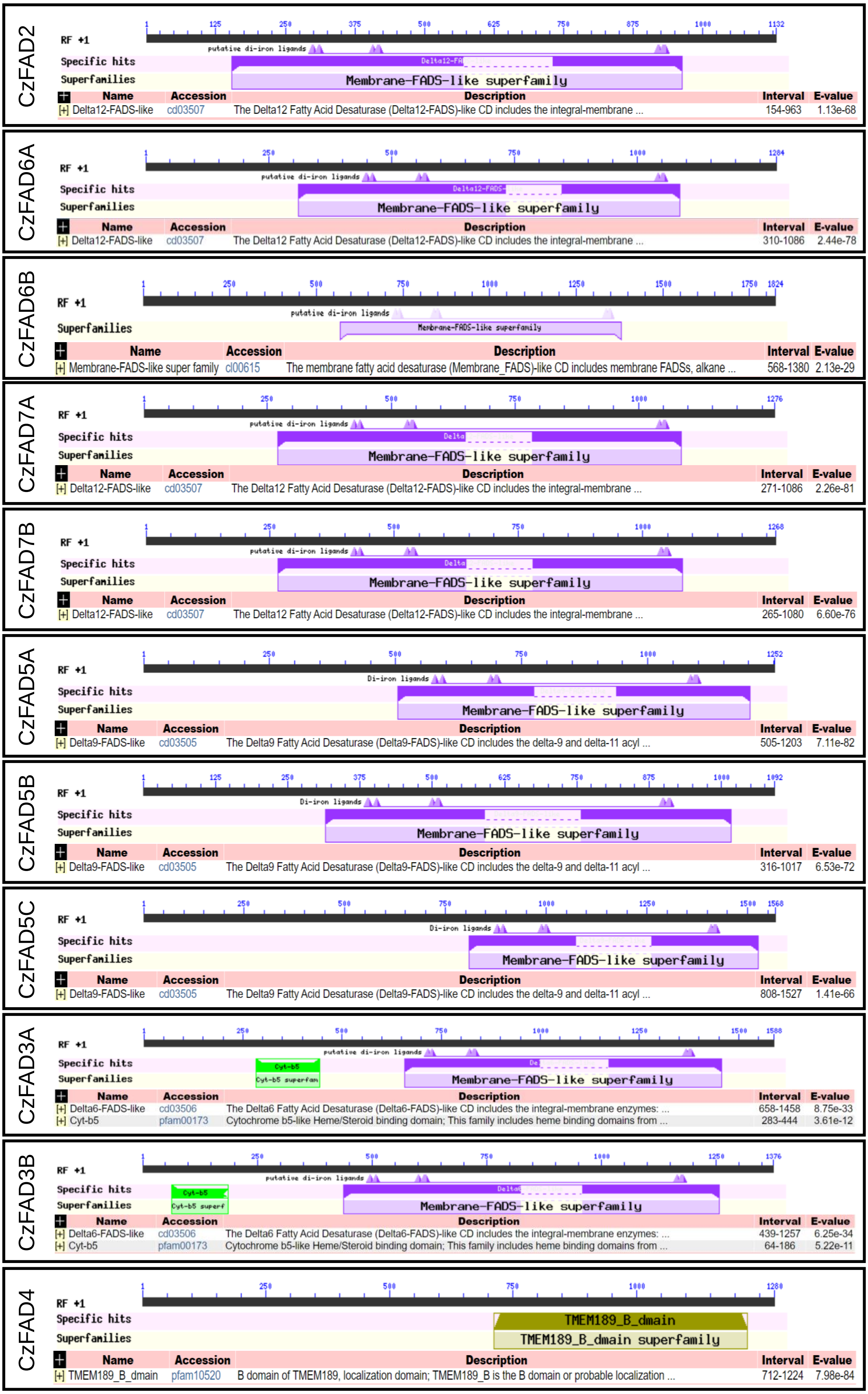

Figure S3. Conserved domains detected in CzFADs by NCBI Conserved Domains Search

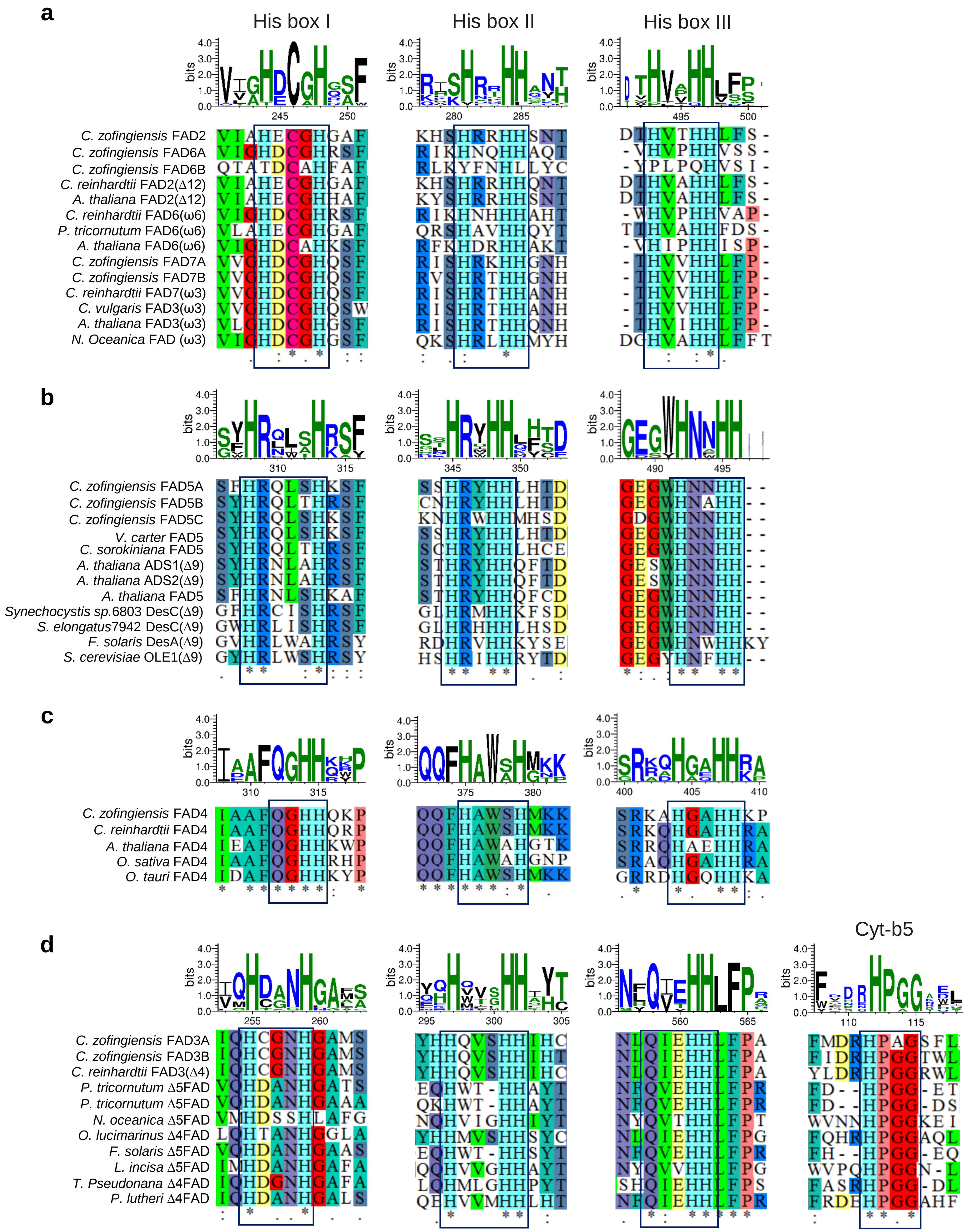

Figure S4. Sequence logo and alignment of functional motifs of Δ12, ω6 and ω3 FADs (a), Δ7/Δ9 FADs (b), $\Delta 3^{trans}$ FADs (c), and front-end FADs (d).

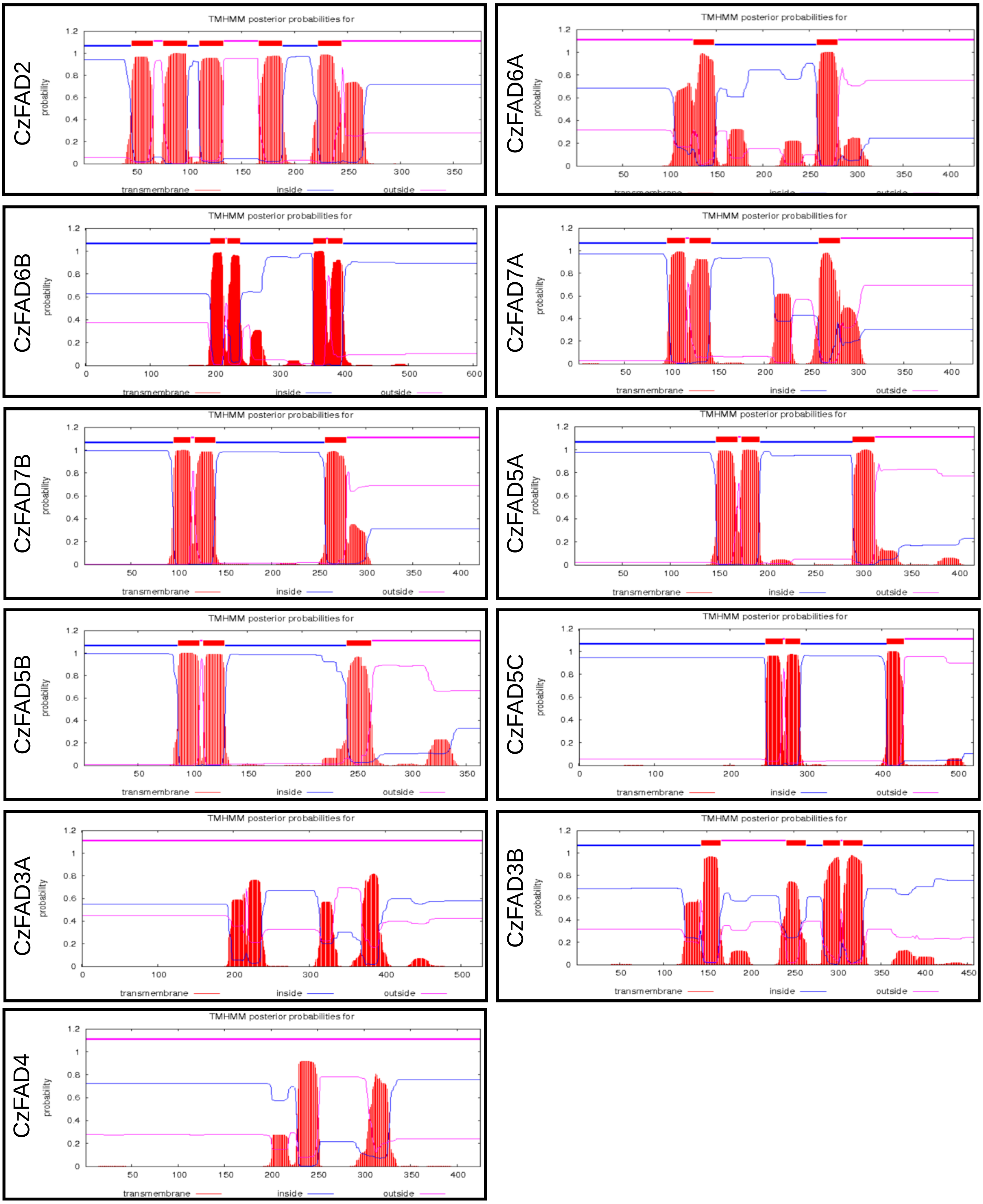

Figure S5. Predicated transmembrane domains for CzFADs by TMHMM (V2.0, http://www.cbs.dtu.dk/services/TMHMM/)

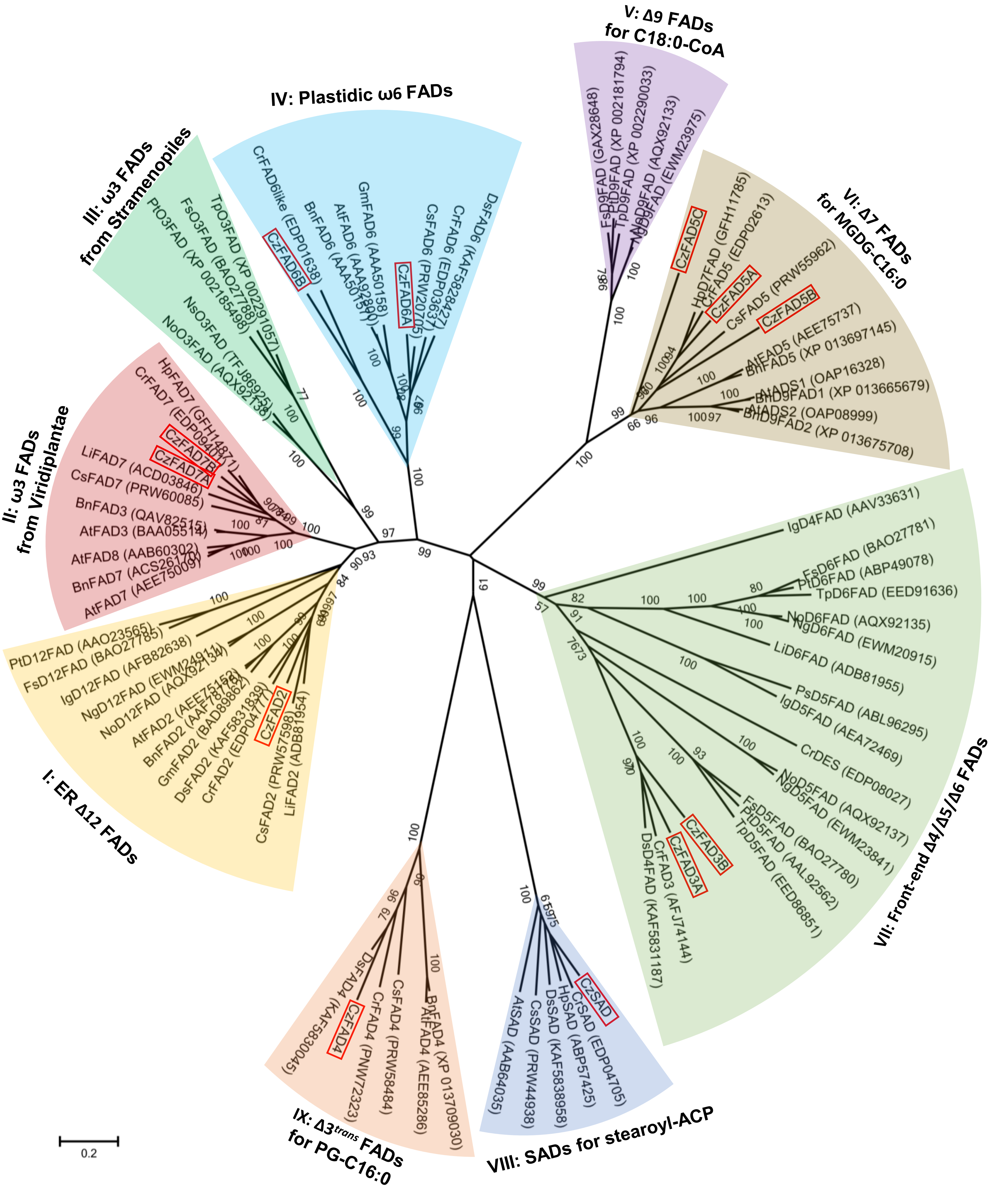

Figure S6. Cladogram of fatty acid desaturases of difference functions from various organisms. The neighbor-joining method was used to reconstruct the cladogram under the software MEGA6, with the bootstrap value (obtained from 1,000 replicates) shown on each node. The scale bar 0.2 represents 20% divergence, calculated as the estimated number of replacement. The GenBank IDs are indicated in the brackets. D and O designate delta (Δ) and omega (ω), respectively. At, *Arabidopsis thaliana*; Bn, *Brassica napus*; Cr, *Chlamydomonas reinhardtii*; Cs, *Chlorella sorokiniana*; Ds, *Dunaliella salina*; Fs, *Fistulifera solaris*; Gm, *Glycine max*; Hp, *Haematococcus pluvialis*; Ig, *Isochrysis galbana*; Li, *Lobosphaera incisa*; Ng, *Nannochloropsis gaditana*; No, *Nannochloropsis oceanica*; Ns, *Nannochloropsis salina*; Ps, *Pavlova salina*; Pt, *Phaeodactylum tricornutum*; Tp, *Thalassiosira pseudonana*.

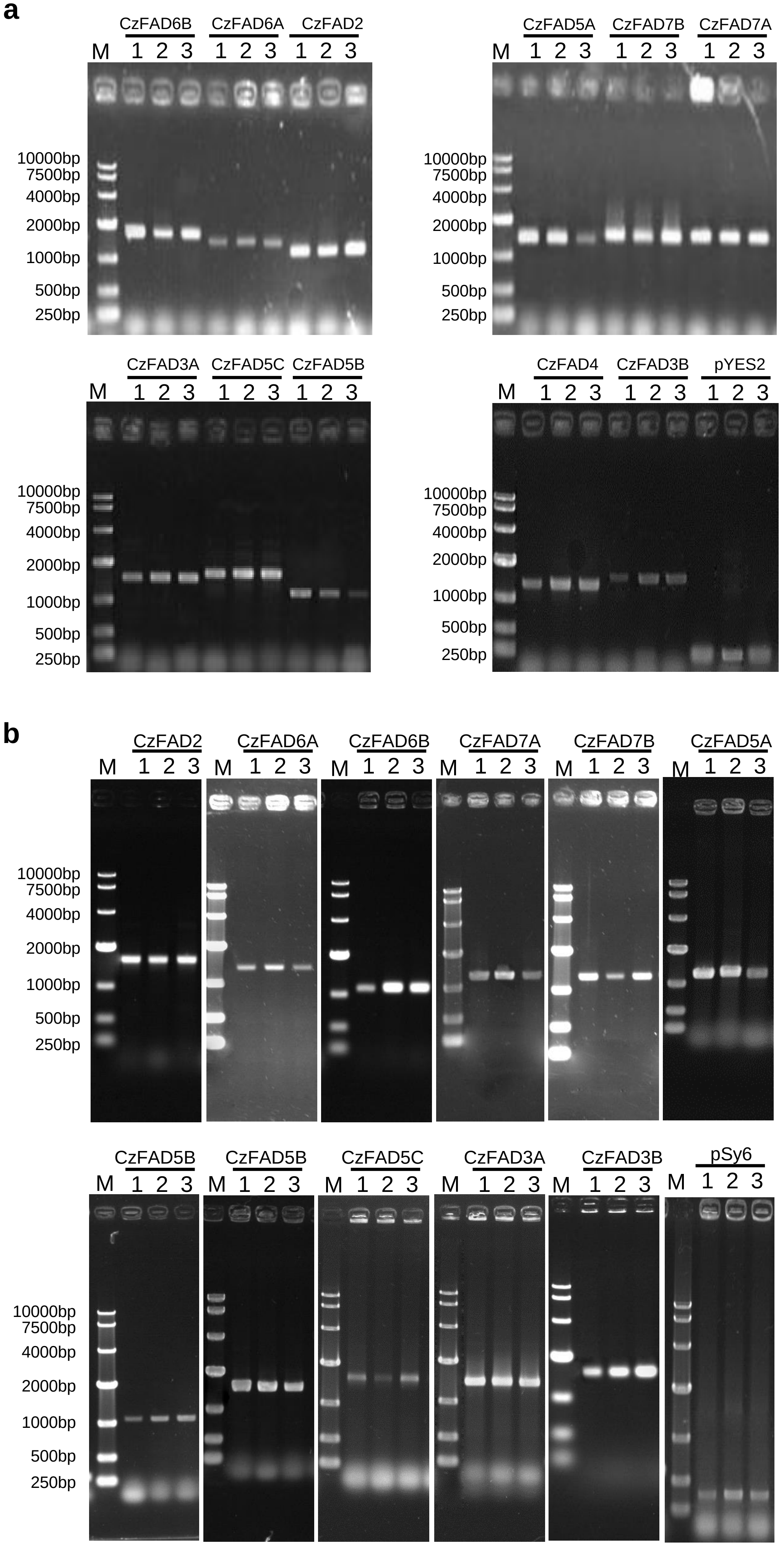

Figure S7. PCR characterization of the *S. cerevisiae* transformants (a) and *S. elongatus* transformants (b) harboring individual *CzFAD* genes

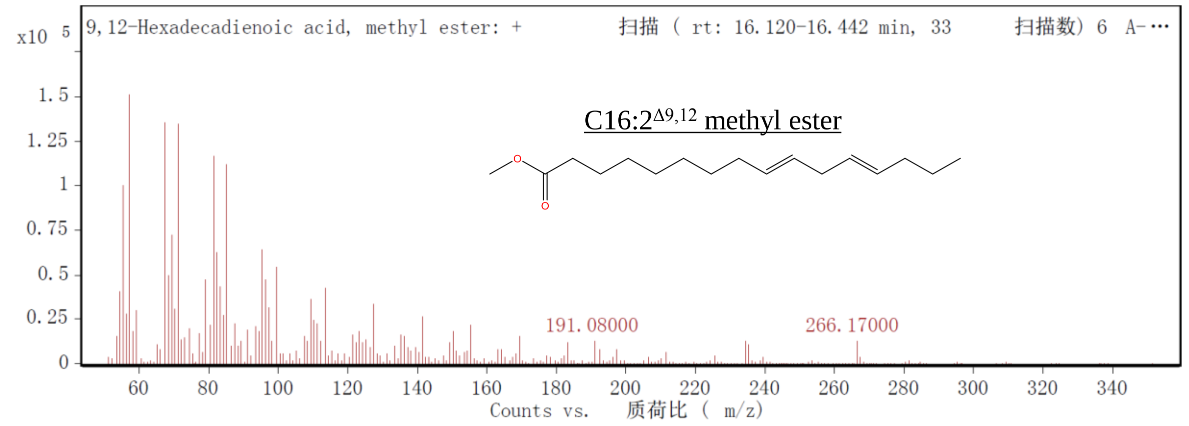
9, 12-Hexadecadienoic acid, methyl ester: +
扫描 (rt: 16. 120-16. 442 min, 33 扫描数) 6 A-…
x10 5
C16:2Δ9,12 methyl ester
191. 08000
266. 17000
Counts vs. 质荷比 (m/z)

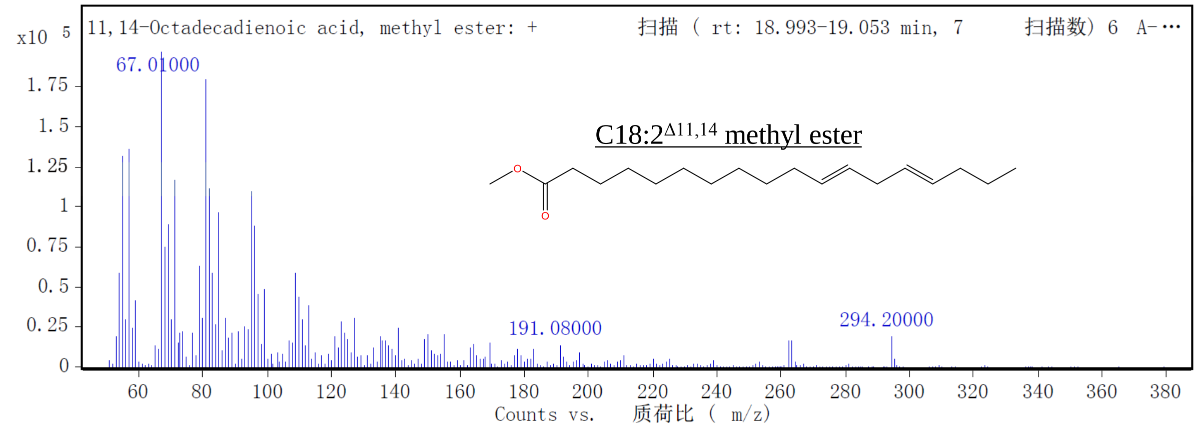
11, 14-Octadecadienoic acid, methyl ester: +
扫描 (rt: 18. 993-19. 053 min, 7 扫描数) 6 A-…
x10 5
67. 01000
C18:2Δ11,14 methyl ester
191. 08000
294. 20000
Counts vs. 质荷比 (m/z)

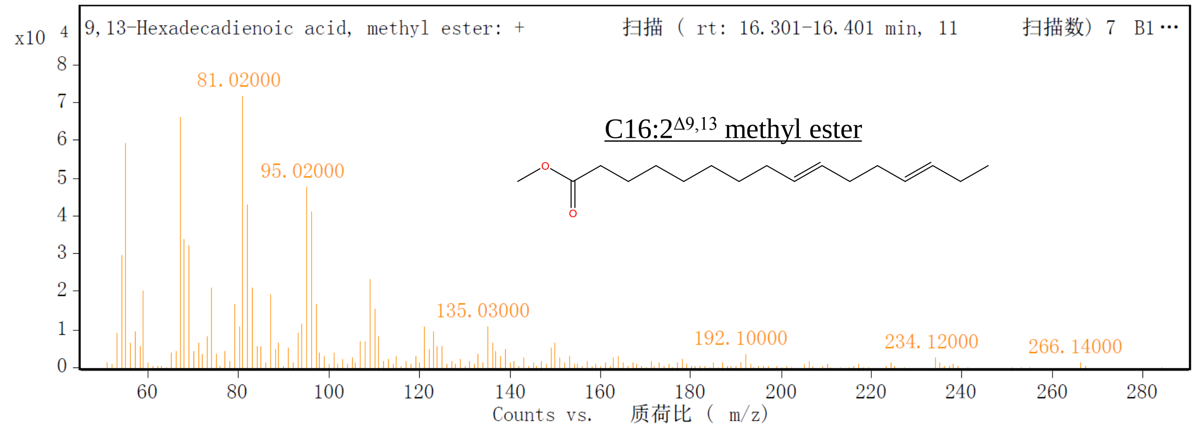
9, 13-Hexadecadienoic acid, methyl ester: +
扫描 (rt: 16. 301-16. 401 min, 11 扫描数) 7 B1…
x10 4
81. 02000
95. 02000
C16:2Δ9,13 methyl ester
135. 03000
192. 10000
234. 12000
266. 14000
Counts vs. 质荷比 (m/z)

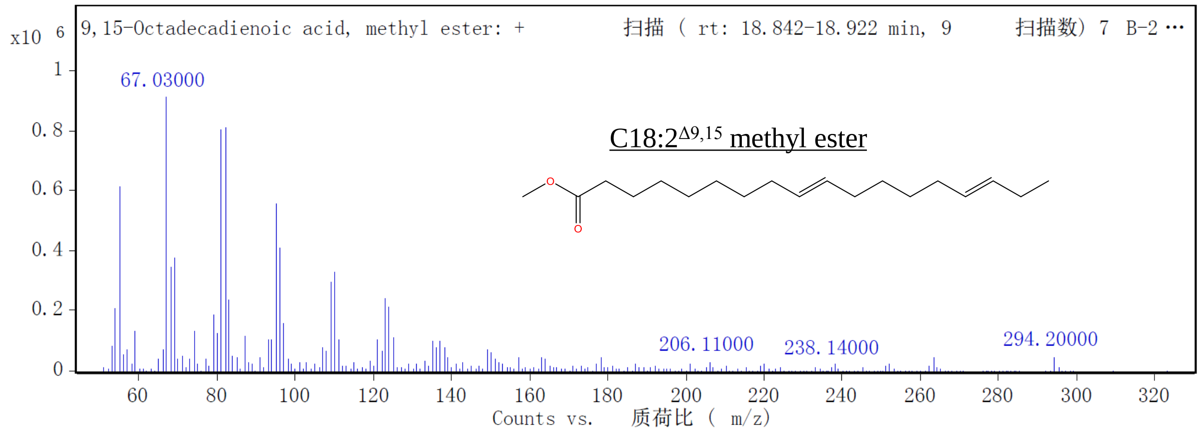
9, 15-Octadecadienoic acid, methyl ester: +
扫描 (rt: 18. 842-18. 922 min, 9 扫描数) 7 B-2…
x10 6
67. 03000
C18:2Δ9,15 methyl ester
206. 11000
238. 14000
294. 20000
Counts vs. 质荷比 (m/z)

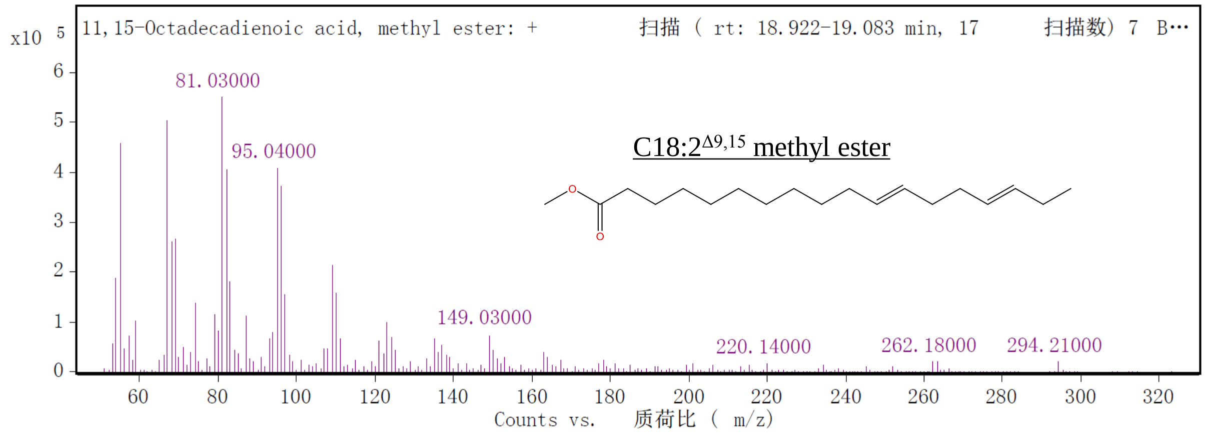
11, 15-Octadecadienoic acid, methyl ester: +
扫描 (rt: 18. 922-19. 083 min, 17 扫描数) 7 B…
x10 5
81. 03000
95. 04000
C18:2Δ9,15 methyl ester
149. 03000
220. 14000
262. 18000
294. 21000
Counts vs. 质荷比 (m/z)

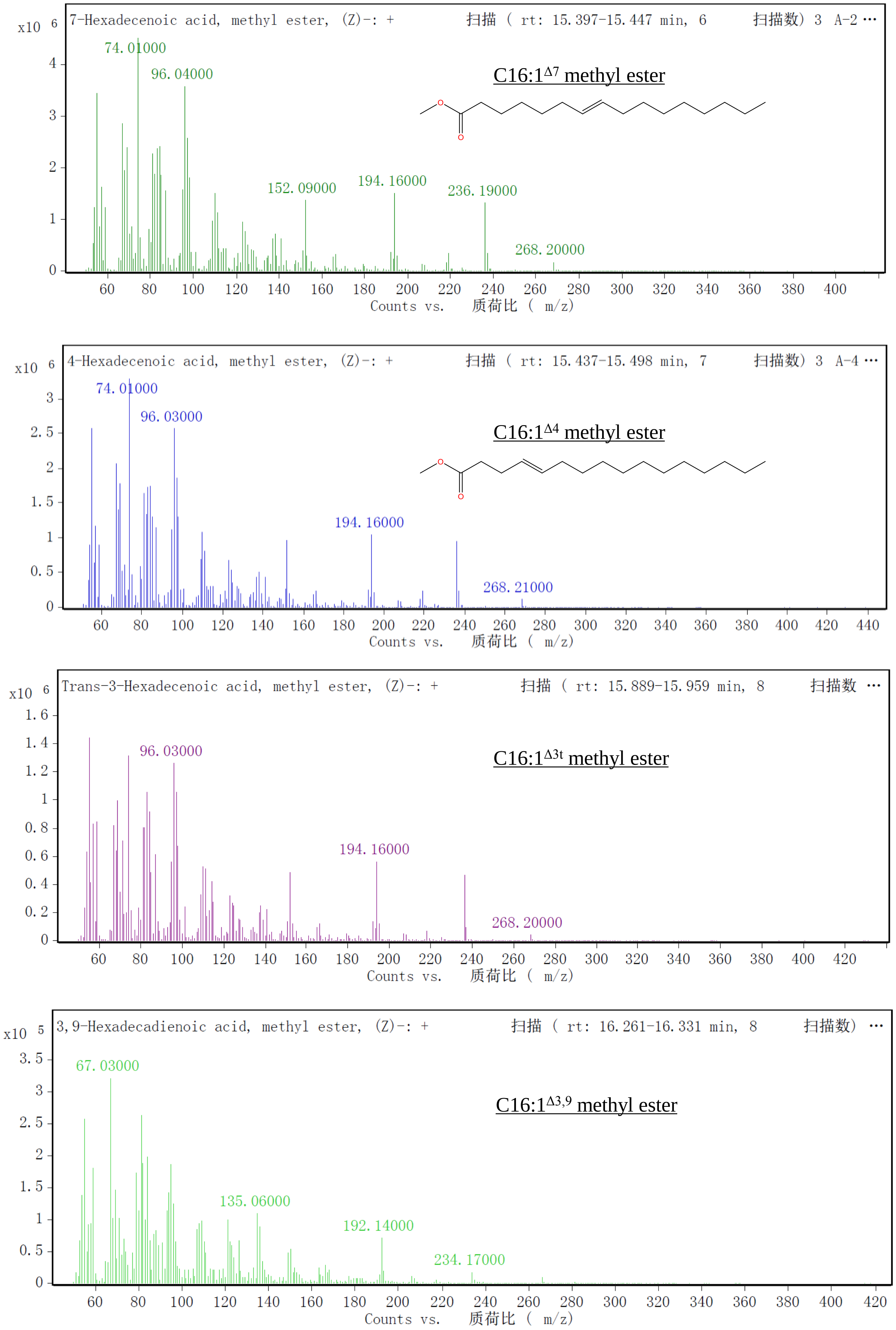

Figure S8. The mass spectra of unusual fatty acids (in the form of methyl ester) produced in transformed *S. elongatus*.

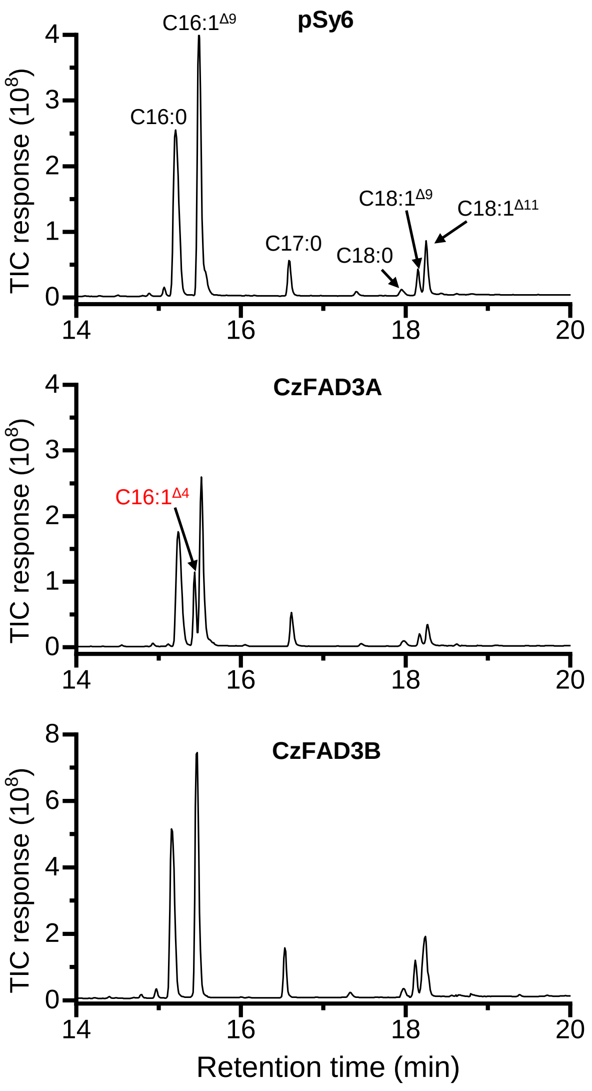

Figure S9. GC-MS chromatography of fatty acids from *S. elongatus* expressing the empty vector pSy6, *CzFAD3A*, or *CzFAD3B.* Newly synthesized fatty acid is designated in red.

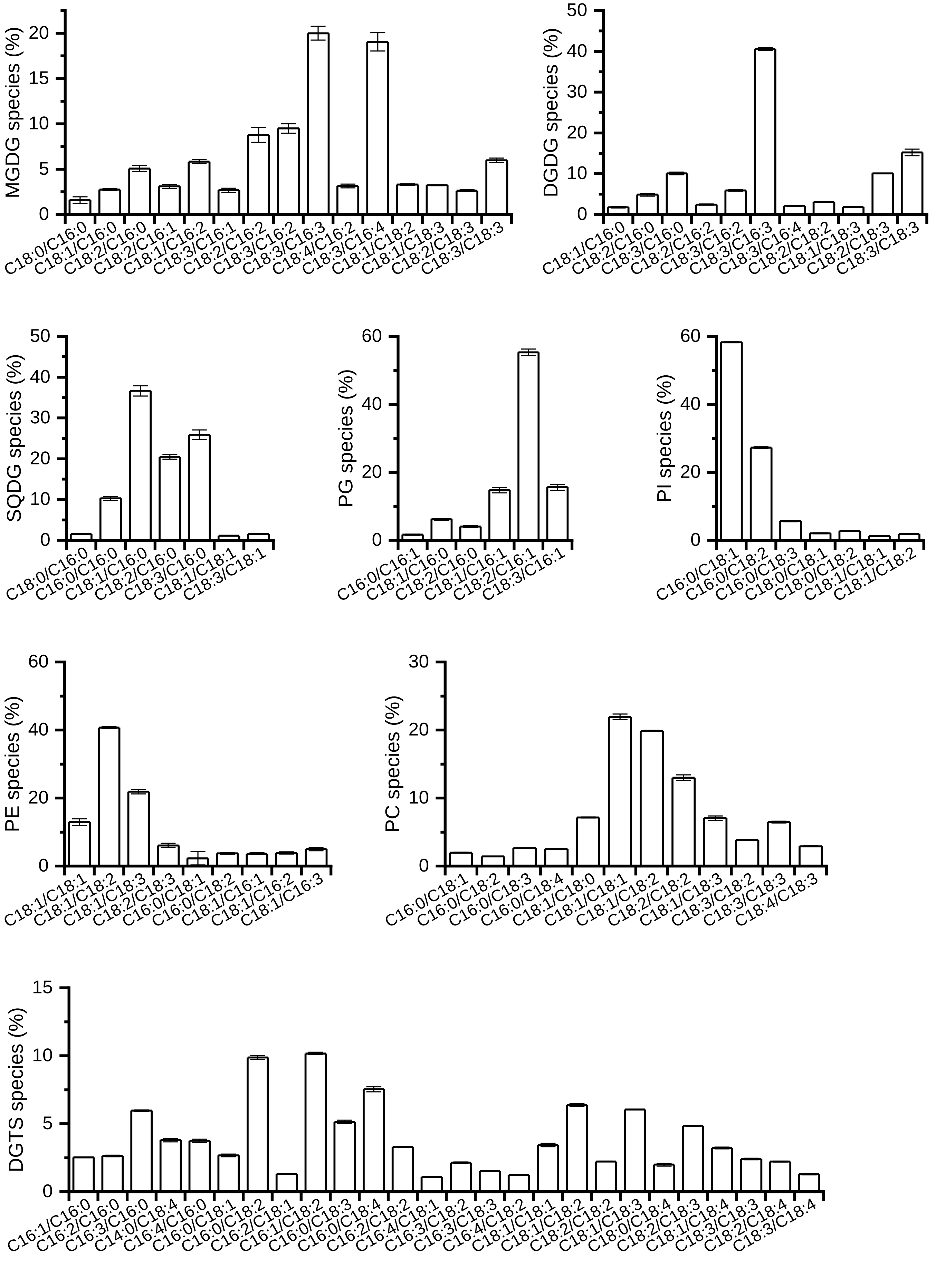

Figure S10. Relative abundance of species of membrane lipid classes in *C. zofingiensis* under favorable growth conditions.

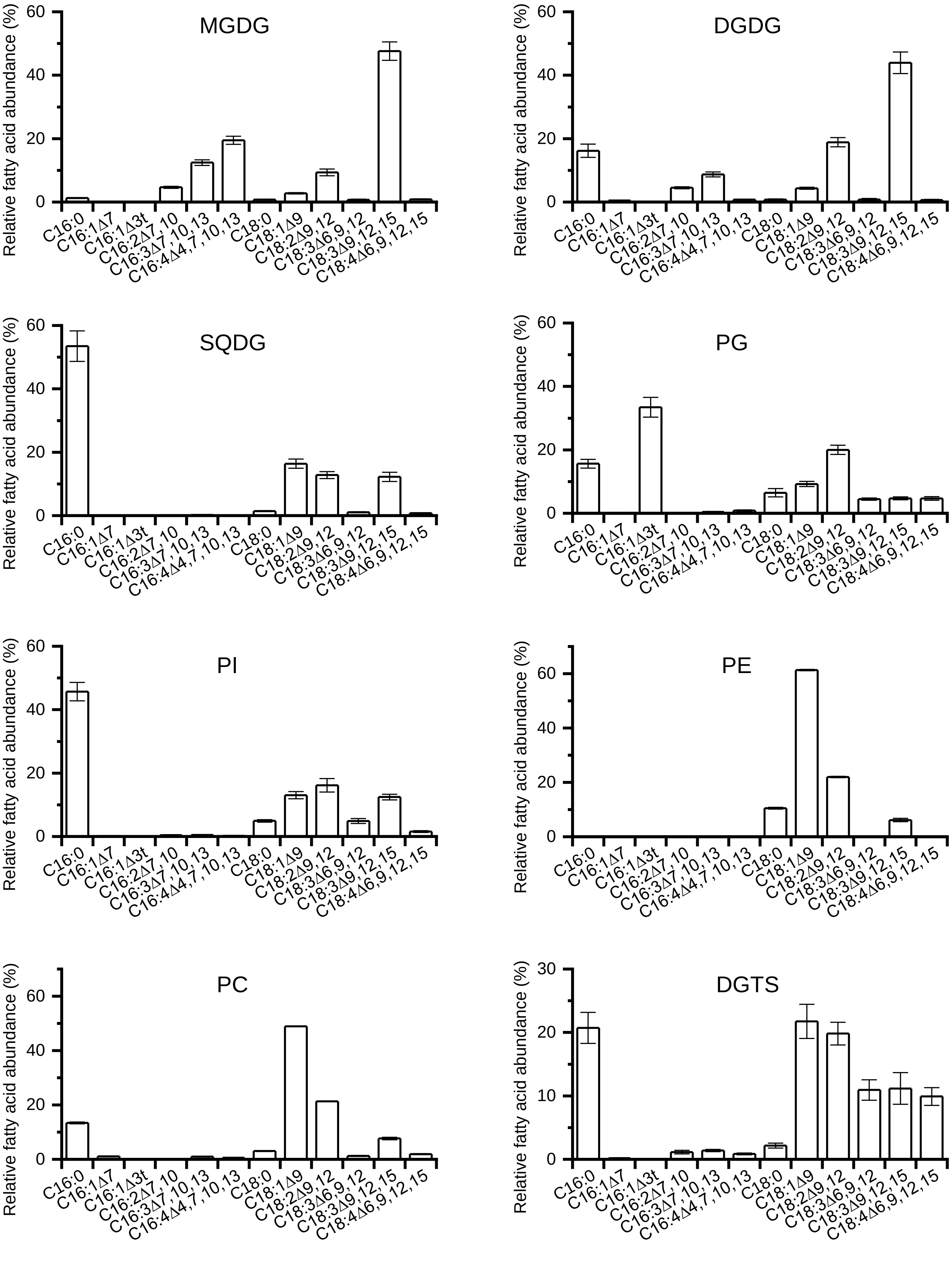

Figure S11. Fatty acid relative abundance of individual membrane lipid classes in *C. zofingiensis* under favorable growth conditions.

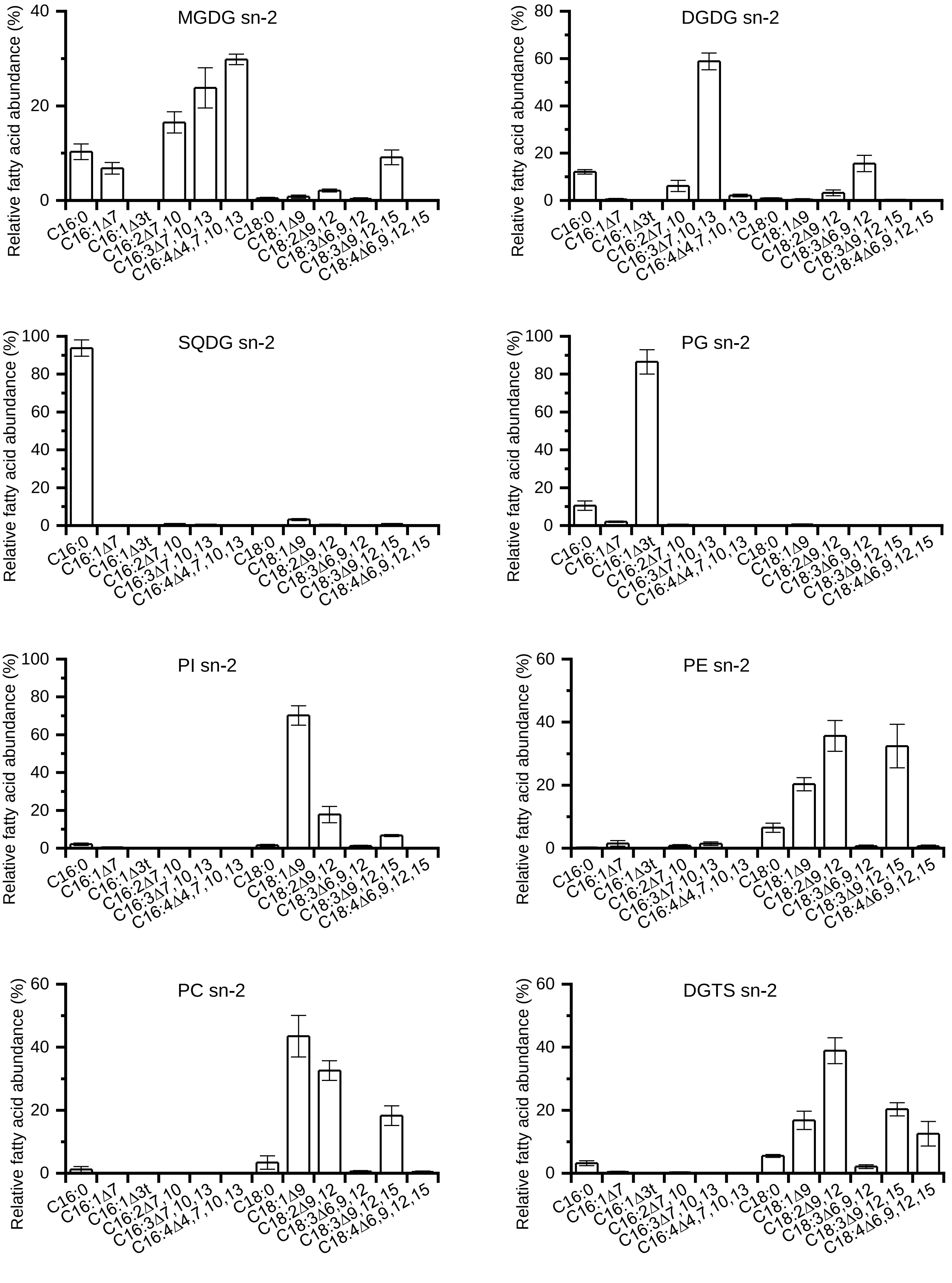

Figure 12. Fatty acid relative abundance of *sn*-2 position of individual membrane lipid classes in *C. zofingiensis* under favorable growth conditions.
