# Supplementary material for: Characterization of fatty acid desaturases reveals stress-induced synthesis of C18 unsaturated fatty acids enriched in triacylglycerol in the oleaginous alga Chromochloris zofingiensis
Source: Biotechnol Biofuels. 2021 Sep 17;14:184. doi: 10.1186/s13068-021-02037-2 (PMC8447527; doi:10.1186/s13068-021-02037-2)
Supplement: Supplementary file 2 — Additional file 2: Table S1. Sequence features of FAD genes in C. zofingiensis. Table S2. Primers used for constructing CzFADs-containing yeast expression vectors. Table S3. Primers used for 5′ RACE experiments of CzFAD genes. Table S4. Primers used for constructing CzFADs-containing S. elongatus expression vectors. Table S5. Primers used for RT-qPCR of CzFAD genes. Table S6. Fatty acid composition of TFA in C. zofingiensis as affected by various stress conditions of ND, SD and SS. Table S7. Fatty acid composition of TAG in C. zofingiensis as affected by various stress conditions of ND, SD and SS. [file 13068_2021_2037_MOESM2_ESM.pdf]

Table S1. Sequence features of *FAD* genes in *C. zofingiensis*

Gene name	Phytozome ID	Genbank ID	ORF (bp)	5'UTR (bp)	Intron	Exon	Subcellular localization		
							Predalgo	TargetP	WoLF PSORT
CzSAD	Cz04g09090	GQ996719	1251	76	6	7	C	C	C
CzFAD2	Cz03g33220	MT323105	1131	44	4	5	O	O	ER
CzFAD6A	Cz08g04110	MT323106	1281	63	8	9	C	C	C
CzFAD6B	Cz11g21120<br>Cz11g21110	MT323107	1821	442	2	3	O	O	C
CzFAD7A	Cz04g31180	MT323108	1275	41	6	7	C	C	C
CzFAD7B	Cz06g28130	MT323109	1266	54	6	7	C	C	C
CzFAD5A	Cz07g00120	MT323110	1251	144	2	3	C	C	C
CzFAD5B	Cz06g00170	MT323111	1092	283	3	4	O	O	C
CzFAD5C	Cz13g01140	MT323112	1566	222	4	5	C	C	C
CzFAD3A	Cz06g12050	MT323113	1587	124	5	6	C	C	C
CzFAD3B	UNPLg00012	MT323114	1374	158	4	5	O	O	ER
CzFAD4	Cz12g10220<br>Cz12g10230	MT323115	1278	46	2	3	C	C	C

ER, endoplasmic reticulum; C, chloroplast; O, others; ORF, open reading frame; SAD, stearoyl-ACP desaturase; UTR, untranslated region.

Table S2. Primers used for constructing *CzFAD*s-containing yeast expression vectors

Primer name	Primer sequence（5′-3′）
CzFAD2-pYES2/CT-F	actatagggaatattaagcttATGGGCAACATGGCCGCT
CzFAD2-pYES2/CT-R	gaagggccctctagactcgagTTAGTCATCCTTGCCTACAGTCTGG
CzFAD6A-pYES2/CT-F	actatagggaatattaagcttATGGCTCAGCTACAGATGGCC
CzFAD6A-pYES2/CT-R	gaagggccctctagactcgagCTACAAGGCACTAGGGATCACTTT
CzFAD6B-pYES2/CT-F	actatagggaatattaagcttATGCATGCAGCAAGAAGTCGC
CzFAD6B-pYES2/CT-F1	actatagggaatattaagcttATGCATTCCAAGAATCATGCACC
CzFAD6B-pYES2/CT-F2	actatagggaatattaagcttATGCAGGGGTTGGATTATGATCAGG
CzFAD6B-pYES2/CT-R1	gaagggccctctagactcgagCTAGCTAAGAACAACAGTGC
CzFAD6B-pYES2/CT-R2	gaagggccctctagactcgagTCAACTGCCGGCTGCGGC
CzFAD7A-pYES2/CT-F	actatagggaatattaagcttATGCAGACTTCACTCTCACGCA
CzFAD7A-pYES2/CT-R	gaagggccctctagactcgagTCAAGCAGATCCTGAAATGCTG
CzFAD7B-pYES2/CT-F	actatagggaatattaagcttATGAACGCCGTCCGCAGC
CzFAD7B-pYES2/CT-R	gaagggccctctagactcgagTTATGCTGTCTTGGCTGTCTGC
CzFAD5A-pYES2/CT-F	actatagggaatattaagcttATGGCTACCGGACTGCTTCA
CzFAD5A-pYES2/CT-R	gaagggccctctagactcgagTTAAGCCTTGGCAAATGCCA
CzFAD5B-pYES2/CT-F	actatagggaatattaagcttATGTCTTTACTGACAGAAACGATGAAG
CzFAD5B-pYES2/CT-R	gaagggccctctagactcgagCTAATCTGCTTTGGTTGCAGCG
CzFAD5C-pYES2/CT-F	actatagggaatattaagcttATGGTATCGACTACATGTCTGCCTA
CzFAD5C-pYES2/CT-R	gaagggccctctagactcgagCTAAACCCTCAACCGCTCCA
CzFAD3A-pYES2/CT-F	actatagggaatattaagcttATGCTGCTGTGTACAAGTGATGC
CzFAD3A-pYES2/CT-R	gaagggccctctagactcgagTTATAATCTTGCTAACTGAGCTGCAG
CzFAD3B-pYES2/CT-F	actatagggaatattaagcttATGGCTGCTGCAGCTTGC
CzFAD3B-pYES2/CT-R	gaagggccctctagactcgagTTACTTGATCTTGTTGATCAGCAACG
CzFAD4-pYES2/CT-F	actatagggaatattaagcttATGCCCTGCTTCTCAAACAGAC
CzFAD4-pYES2/CT-F1	actatagggaatattaagctt ATGCCCAGCACAGGGTTCGTCA
CzFAD4-pYES2/CT-F2	actatagggaatattaagctt ATGTATTGTCCTCGTCAAGTGCT
CzFAD4-pYES2/CT-R1	gaagggccctctagactcgagTTAGATAGCACCAGTCAGTTTG
CzFAD4-pYES2/CT-R2	gaagggccctctagactcgagTCACATTGAGCTGTCTGGTGCT
GAL1-F	AATATACCTCTATACTTTAACGTC
CYC1-R	GCGTGAATGTAAGCGTGAC

F, forward; R, reverse

Table S3. Primers used for 5′ RACE experiments of *CzFAD* genes

Gene name	Primer sequence（5′-3′）	
	1st round RACE primer	2nd RACE primer
CzFAD2	CCAGGAATAGTAAGGCACCAGCAGCG	AGGCGTAGAACAGAGCCGCAGCA
CzFAD6A	CTGGCAGAGAAGGACGCAATGGAC	CCTTGCTTCGTCTGACAGCTGTGAG
CzFAD6B	CTGCTTGGGGAGTCTGTGGGGCT	GCTAGGGCTGTGTTCAGGTCGATGG
CzFAD7A	AGCAAGCCCTGCCACAATAGCCA	GCGGGGCTGCTGACAAGTCGTAA
CzFAD7B	CTGATAAGCTGAAAGGTGGCGGGG	TTGAAAGCCTCTGTCATCCACTAGG
CzFAD5A	GGTTCCTGTGCTTGAGCCACTTTGC	CAAGCGCCTGGCACCTGATTGTC
CzFAD5B	CTGCGTTGACATTGTGGCTGGTTCT	GCAAGAAGCAAAGGCCTCTGCTTG
CzFAD5C	GGTGGGTCTGTGCTGACGAGGCTT	GCGTTGGGGGCTTGTGAAAGGTATC
CzFAD3A	CCATTTACTGTCCTCCCAGGGGTCTTG	CATTGCGCTGGTGCTTGAAGTGACT
CzFAD3B	TGTCATTTGGGTATGGAGCACGTGG	TCTCTGCCTACTGCCAGGTTGATGA
CzFAD4	CGGCTGTGCTGGCTAAAAGTTCATTC	CCTCCTCCCGCACCTCCTCCATAAG

Table S4. Primers used for constructing *CzFAD*s-containing *S. elongatus* expression vectors

Primer name	Primer sequence （5′-3′）
CzFAD2-pSyn6-F	ctgtactttcagggcaagcttATGGGCAACATGGCCGCT
CzFAD2-pSyn6-R	tttgcctggtaccgcggatccTTAGTCATCCTTGCCTACAGTCTGG
CzFAD6A-pSyn6-F	ctgtactttcagggcaagcttATGGCTCAGCTACAGATGGCC
CzFAD6A-pSyn6-R	tttgcctggtaccgcggatccCTACAAGGCACTAGGGATCACTTTC
CzFAD6B-pSyn6-F	ctgtactttcagggcaagcttATGCATGCAGCAAGAAGTCGC
CzFAD6B-pSyn6-R	tttgcctggtaccgcggatccTCAACTGCCGGCTGCGGC
CzFAD7A-pSyn6-F	ctgtactttcagggcaagcttATGCAGACTTCACTCTCACGCA
CzFAD7A-pSyn6-R	tttgcctggtaccgcggatccTCAAGCAGATCCTGAAATGCTG
CzFAD7B-pSyn6-F	ctgtactttcagggcaagcttATGAACGCCGTCCGCAGC
CzFAD7B-pSyn6-R	tttgcctggtaccgcggatccTTATGCTGTCTTGGCTGTCTGC
CzFAD5A-pSyn6-F	ctgtactttcagggcaagcttATGGCTACCGGACTGCTTCA
CzFAD5A-pSyn6-R	tttgcctggtaccgcggatccTTAAGCCTTGGCAAATGCCA
CzFAD5B-pSyn6-F	ctgtactttcagggcaagcttATGTCTTTACTGACAGAAACGATGAAG
CzFAD5B-pSyn6-R	tttgcctggtaccgcggatccCTAATCTGCTTTGGTTGCAGCG
CzFAD5C-pSyn6-F	ctgtactttcagggcaagcttATGGTATCGACTACATGTCTGCCTA
CzFAD5C-pSyn6-R	tttgcctggtaccgcggatccCTAAACCCTCAACCGCTCCA
CzFAD3A-pSyn6-F	ctgtactttcagggcaagcttATGCTGCTGTGTACAAGTGATGC
CzFAD3A-pSyn6-R	tttgcctggtaccgcggatccTTATAATCTTGCTAACTGAGCTGCAG
CzFAD3B-pSyn6-F	ctgtactttcagggcaagcttATGGCTGCTGCAGCTTGC
CzFAD3B-pSyn6-R	tttgcctggtaccgcggatccTTACTTGATCTTGTTGATCAGCAACG
CzFAD4-pSyn6-F	ctgtactttcagggcaagcttATGCCCTGCTTCTCAAACAGAC
CzFAD4-pSyn6-R	tttgcctggtaccgcggatccTCACATTGAGCTGTCTGGTGCT
pSyn6-F	AGGGTTTTTACACCTTTTTGAC
pSyn6-R	GGGACCACCGCGCTACTGCCG

F, forward; R, reverse

Table S5. Primers used for RT-qPCR of *CzFAD* genes

Gene name	Forward（5′-3′）	Reverse（5′-3′）
CzSAD	GATGAGGGACGGCATGAAAT	GTGTGCGGGCATCACAATC
CzFAD2	CTTGCTGCGGCTCTGTTCTA	ACAACACCCTGCCAGAACCA
CzFAD6A	GACTTGGCTTCCCCGCTATT	GCGCCGTGTGATGTACAACT
CzFAD6B	TCATGGCACCTTCATTGTTCA	AGGTCGCTGGTCCGTATGG
CzFAD7A	CCACCATTGACAGGGATTACG	CCTCTGTGGCTTCCTCCAAA
CzFAD7B	CCATGTTCTGGGCGTTGTTT	AGCCGTGGTATGGTACAAGGA
CzFAD5A	CCAGGTCTGTTGTGCGTGTT	TGCTGGTCGTGTGCTTGTGT
CzFAD5B	TCTGCCGAAGCCATCCAT	CGTTGACATTGTGGCTGGTT
CzFAD5C	CAAGTGTCCCCCATCCAAAT	TGTCACCAAAATAAGGCCGTTT
CzFAD3A	GCATTCCAGGTGGGAGACAT	CCAGGGTCAGGGTGTAGTGAA
CzFAD3B	CTGTTGCTGCTTATGCCCTGTA	TGAGCGGGTTGTTGGACATT
CzFAD4	CAGCAGCGTCAATCCAACAG	GCTGCCATAAGTCCCATTGTC
CzActin	GCTGGCATTCACGACACAAC	TGCCACCACCTTGATCTTCA

Table S6. Fatty acid composition of TFA in *C. zofingiensis* as affected by various stress conditions of ND, SD and SS

% of TFA	Day 0	Day 2			Day 4			Day 6		
	Control	ND	SD	SS	ND	SD	SS	ND	SD	SS
C16:0	19.06 ±0.26	15.29 ±0.03	19.81 ±0.38	18.05 ±0.34	15.17 ±0.08	21.02 ±0.10	17.77 ±0.21	14.68 ±0.20	23.63 ±0.36	18.10 ±0.67
$C16:1^{\Delta 7}$	3.92 ±0.15	2.86 ±0.05	3.59 ±0.05	3.00 ±0.02	3.84 ±0.02	5.45 ±0.07	4.09 ±0.07	3.01 ±0.05	5.33 ±0.04	3.40 ±0.03
$C16:1^{\Delta 3t}$	2.38 ±0.34	–	–	2.16 ±0.11	–	–	2.15 ±0.11	–	–	–
$C16:2^{\Delta 7,10}$	4.75 ±0.17	2.94 ±0.29	2.95 ±0.14	3.08 ±0.12	2.73 ±0.06	2.62 ±0.10	3.33 ±0.47	3.93 ±0.04	3.16 ±0.05	4.51 ±0.46
$C16:3^{\Delta 7,10,13}$	7.02 ±0.49	6.02 ±0.69	6.03 ±0.09	8.07 ±0.45	6.22 ±0.65	6.00 ±0.17	6.33 ±0.15	5.71 ±0.14	5.45 ±0.18	6.74 ±0.69
$C16:4^{\Delta 4,7,10,13}$	4.73 ±0.14	0.62 ±0.06	0.89 ±0.05	1.49 ±0.16	0.43 ±0.03	0.63 ±0.05	1.71 ±0.19	0.28 ±0.01	0.48 ±0.02	3.39 ±0.06
C18:0	2.41 ±0.03	2.05 ±0.20	3.91 ±0.22	2.96 ±0.34	2.56 ±0.23	4.36 ±0.12	2.62 ±0.07	3.05 ±0.14	3.43 ±0.19	3.54 ±0.10
$C18:1^{\Delta 9}$	18.83 ±0.34	43.22 ±0.22	39.80 ±0.65	34.17 ±1.12	44.34 ±1.48	40.85 ±1.13	38.80 ±0.39	44.28 ±1.40	42.12 ±2.15	35.87 ±1.26
$C18:2^{\Delta 9,12}$	16.61 ±0.64	18.21 ±0.62	12.29 ±0.08	16.35 ±0.38	17.01 ±0.59	10.59 ±0.05	15.80 ±0.38	18.10 ±0.42	9.12 ±0.24	17.09 ±0.15
$C18:3^{\Delta 6,9,12}$	0.59 ±0.01	0.32 ±0.03	0.28 ±0.02	0.48 ±0.04	0.27 ±0.02	0.22 ±0.01	0.61 ±0.06	0.23 ±0.00	0.19 ±0.01	0.49 ±0.02
$C18:3^{\Delta 9,12,15}$	18.86 ±0.45	8.18 ±0.08	10.08 ±0.15	9.71 ±0.49	7.18 ±0.09	7.87 ±0.20	6.17 ±0.47	6.55 ±0.11	6.64 ±0.05	6.27 ±0.32
$C18:4^{\Delta 6,9,12,15}$	0.84 ±0.02	0.29 ±0.03	0.37 ±0.01	0.48 ±0.08	0.25 ±0.01	0.39 ±0.01	0.62 ±0.04	0.18 ±0.01	0.45 ±0.01	0.60 ±0.05
SFA	21.47 ±0.17	17.34 ±0.16	23.72 ±0.32	21.01 ±0.32	17.73 ±0.12	25.38 ±0.10	20.39 ±0.15	17.73 ±0.17	27.06 ±0.27	21.64 ±0.53
MUFA	25.13 ±0.31	46.08 ±0.18	43.39 ±0.57	39.33 ±0.89	48.18 ±0.46	46.30 ±0.11	45.04 ±0.31	47.29 ±0.38	47.45 ±0.10	39.27 ±0.24
PUFA	53.40 ±0.43	36.58 ±0.45	32.89 ±0.08	39.66 ±0.41	34.09 ±0.38	28.32 ±0.08	34.57 ±0.31	34.98 ±0.23	25.49 ±0.08	39.09 ±0.28
DUS	1.70 ±0.07	1.36 ±0.05	1.28 ±0.04	1.41 ±0.05	1.31 ±0.03	1.19 ±0.01	1.32 ±0.03	1.31 ±0.05	1.13 ±0.03	1.39 ±0.04

SFA, percentage of saturated FAs; MUFA, percentage of monounsaturated Fas; PUFA, percentage of polyunsaturated Fas; DUS: degree of fatty acid unsaturation = [1.0 (% monoenes) + 2.0 (% dienes) + 3.0 (% trienes) + 4.0 (% tetraenes)]/100

Table S7. Fatty acid composition of TAG in *C. zofingiensis* as affected by various stress conditions of ND, SD and SS

% of TAG-FA	Day 0	Day 2			Day 4			Day 6		
	Control	ND	SD	SS	ND	SD	SS	ND	SD	SS
C16:0	29.25 ±0.02	21.11 ±0.13	27.47 ±1.78	21.45 ±0.53	21.12 ±0.19	23.37 ±0.84	23.13 ±0.58	19.39 ±0.50	23.37 ±0.30	22.79 ±0.30
$C16:1^{\Delta 7}$	1.82 ±0.35	1.92 ±0.07	3.58 ±0.32	1.68 ±0.18	2.98 ±0.43	5.73 ±0.64	1.73 ±0.05	2.72 ±0.81	7.31 ±0.51	1.77 ±0.10
$C16:2^{\Delta 7,10}$	1.91 ±0.24	2.01 ±0.15	1.49 ±0.04	1.22 ±0.08	2.40 ±0.03	1.95 ±0.17	0.89 ±0.00	3.64 ±0.02	2.31 ±0.05	1.50 ±0.07
$C16:3^{\Delta 7,10,13}$	1.02 ±0.18	1.81 ±0.18	1.09 ±0.04	1.34 ±0.15	1.61 ±0.05	1.39 ±0.21	1.08 ±0.02	2.08 ±0.18	1.86 ±0.08	2.57 ±0.41
$C16:4^{\Delta 4,7,10,13}$	2.93 ±0.63	0.35 ±0.04	0.20 ±0.02	0.56 ±0.07	0.25 ±0.01	0.26 ±0.04	0.38 ±0.10	0.30 ±0.05	0.34 ±0.04	0.92 ±0.11
C18:0	12.70 ±2.16	–	–	4.08 ±0.16	–	–	5.20 ±0.12	–	–	5.46 ±0.17
$C18:1^{\Delta 9}$	33.37 ±0.89	46.03 ±1.46	47.40 ±1.49	46.80 ±1.05	49.94 ±0.32	46.98 ±0.70	45.69 ±1.74	44.97 ±0.19	46.41 ±0.63	45.05 ±0.83
$C18:2^{\Delta 9,12}$	9.82 ±1.60	17.90 ±0.85	11.91 ±0.71	14.37 ±0.28	14.49 ±0.50	13.68 ±0.88	13.94 ±0.27	19.48 ±0.30	12.24 ±0.46	12.53 ±0.54
$C18:3^{\Delta 6,9,12}$	1.27 ±0.26	0.42 ±0.01	0.11 ±0.05	0.43 ±0.02	0.40 ±0.09	0.22 ±0.03	0.29 ±0.01	0.55 ±0.08	0.23 ±0.04	0.45 ±0.06
$C18:3^{\Delta 9,12,15}$	4.35 ±0.42	8.22 ±0.42	6.49 ±0.76	7.09 ±0.18	6.61 ±0.25	6.15 ±0.81	6.85 ±0.07	6.68 ±0.68	5.62 ±0.07	6.41 ±0.15
$C18:4^{\Delta 6,9,12,15}$	0.66 ±0.29	0.17 ±0.03	0.12 ±0.04	0.29 ±0.03	0.16 ±0.00	0.18 ±0.06	0.16 ±0.01	0.19 ±0.04	0.31 ±0.02	0.41 ±0.08
SFA	41.95 ±1.07	21.11 ±0.13	27.47 ±1.78	25.53 ±0.56	21.12 ±0.19	23.37 ±0.84	28.33 ±0.43	19.39 ±0.50	23.37 ±0.30	28.25 ±0.19
MUFA	36.09 ±0.86	48.01 ±1.52	51.12 ±1.52	49.17 ±1.04	52.96 ±0.29	52.80 ±0.66	48.08 ±1.66	47.69 ±0.12	53.72 ±0.55	46.96 ±0.76
PUFA	21.96 ±0.97	30.88 ±0.42	21.41 ±0.72	25.30 ±0.28	25.92 ±0.32	23.83 ±0.58	23.59 ±0.22	32.92 ±0.52	22.91 ±0.10	24.79 ±0.09
DUS	0.94 ±0.03	1.21 ±0.07	1.02 ±0.03	1.10 ±0.04	1.14 ±0.01	1.09 ±0.03	1.05 ±0.04	1.24 ±0.06	1.09 ±0.02	1.09 ±0.03

SFA, percentage of saturated FAs; MUFA, percentage of monounsaturated Fas; PUFA, percentage of polyunsaturated Fas; DUS: degree of fatty acid unsaturation = [1.0 (% monoenes) + 2.0 (% dienes) + 3.0 (% trienes) + 4.0 (% tetraenes)]/100
